# AdaBoost-based differential diagnosis of gouty arthritis and rheumatoid arthritis: model construction, temporal validation, and SHAP visualization

**DOI:** 10.3389/fmed.2026.1855696

**Published:** 2026-08-10

**Authors:** Jing Su, Tao Peng, Zilin Chen, Xuchun Ou, Qiling Deng, Xin Ding, Peiqi Liang, Jianhua Xu

**Affiliations:** 1 Department of Laboratory Medicine, The Third Affiliated Hospital of Guangzhou University of Chinese Medicine, Guangzhou, China; 2 Science and Technology Innovation Center, Guangzhou University of Chinese Medicine, Guangzhou, China; 3 State Key Laboratory of Traditional Chinese Medicine Syndrome/Department of Laboratory Science, The Second Affiliated Hospital of Guangzhou University of Chinese Medicine, Guangdong Provincial Hospital of Chinese Medicine, Guangdong Provincial Academy of Chinese Medical Sciences, Guangzhou, China

**Keywords:** Adaboost, differential diagnosis, gouty arthritis, immunometabolism, machine learning, rheumatoid arthritis, SHAP analysis

## Abstract

**Background:**

The phenotypic overlap between gouty arthritis (GA) and Rheumatoid Arthritis (RA) poses diagnostic challenges, particularly in resource-limited settings. This study aims to develop a robust, non-invasive machine learning (ML) framework based on routine hematological parameters to facilitate precise differential diagnosis and to evaluate its robustness in a temporally independent cohort.

**Methods:**

Adopting a temporal validation design, we retrospectively analyzed clinical data from a tertiary hospital. A development cohort (2021–2025; *n* = 9,903) was utilized for model construction, while an independent historical cohort (2015–2020; *n* = 1,983) served as an time-based internal validation set. Feature selection was performed via LASSO regression, followed by benchmarking nine ML algorithms, including AdaBoost. Performance was evaluated using the Area Under the Curve (AUC) and Decision Curve Analysis (DCA), with interpretability enhanced by the SHAP framework.

**Results:**

Six pivotal features were identified, comprising demographics, traditional markers, and novel composite ratios (CHOL/TG). AdaBoost demonstrated superior performance, achieving an AUC of 0.898 in the internal test set and an impressive 0.897 in the time-based internal validation, indicating exceptional generalization. SHAP analysis revealed that beyond conventional markers, CHOL/TG is a key predictor, revealing a unique interaction pattern of lipid metabolism that distinguishes gouty arthritis (GA) from rheumatoid arthritis (RA).

**Conclusion:**

We successfully developed and validated an AdaBoost-based model leveraging routine blood indices for precise GA/RA differentiation. Validated across temporal cohorts, this cost-effective tool holds significant promise for optimizing early triage in primary care, while offering novel pathophysiological insights through the lens of lipid metabolism.

## 1 Introduction

Gouty arthritis (GA) and rheumatoid arthritis (RA) represent the two most prevalent chronic inflammatory arthropathies (1, 2). Despite their distinct pathophysiological underpinnings—stemming from monosodium urate (MSU) crystal deposition-induced inflammatory cascades versus systemic autoimmune-mediated synovitis (3, 4)—they exhibit substantial phenotypic overlap. During acute flares, GA typically manifests with cardinal signs of inflammation—intense erythema, swelling, and pain—classically affecting the first metatarsophalangeal joint (5–7); however, atypical presentations are increasingly recognized. Concurrently, early-stage RA does not invariably follow the classic symmetric polyarticular pattern (8, 9); monoarticular onset is frequently observed at initial presentation, creating a diagnostic “gray zone.” Complicating matters further, chronic tophi in GA can induce joint erosions that radiologically mimic the osteolytic changes characteristic of RA (8, 10). This diagnostic ambiguity poses a significant challenge not only to rheumatologists but also to primary care practitioners, often resulting in substantial diagnostic delays that compromise patient outcomes (11). Misdiagnosis carries severe iatrogenic risks: inappropriate immunosuppression for GA or inadequate urate-lowering therapy for RA can irreversibly deteriorate the patient’s condition (12). Therefore, achieving rapid and precise differentiation at the initial clinical encounter is pivotal for optimizing long-term functional prognosis (13).

While established clinical guidelines provide classification criteria, their implementation in routine practice remains fraught with challenges (14). The diagnostic gold standard—synovial fluid analysis for monosodium urate (MSU) crystals—is hampered by its invasive nature, variable patient compliance, and the requirement for specialized microscopy expertise (15), resulting in limited utilization in outpatient settings. Serologically, serum uric acid levels can be paradoxically normal during acute flares due to the uricosuric effects of inflammation (16). Conversely, seronegativity for rheumatoid factor (RF) and anti-cyclic citrullinated peptide (anti-CCP) antibodies occurs in a subset of RA patients (15), further complicating diagnosis. Given this biochemical non-specificity, traditional statistical models relying on linear assumptions often fail to capture the high-dimensional (17), nonlinear interactions inherent in complex clinical datasets. Machine learning (ML), a pivotal branch of artificial intelligence, offers a paradigm shift in handling such nonlinear medical complexities (18). By integrating demographic profiles, granular hematological parameters, and clinical phenotypes, ML algorithms construct multidimensional predictive matrices to unveil latent associations that may elude human heuristic reasoning. Previous studies in pulmonology and autoimmune diseases have demonstrated that ML approaches consistently outperform conventional scoring systems (19).

Therefore, this study aims to develop and validate a comprehensive ML framework integrating multidimensional clinical and laboratory metrics. We benchmarked nine distinct algorithms—XGBoost, logistic regression, LightGBM, RandomForest, AdaBoost, DecisionTree, GBDT, GNB, and SVM—to identify the optimal model for discriminating GA from RA. Furthermore, we employed SHAP (SHapley Additive exPlanations) to demystify the algorithmic decision-making process, ensuring the provision of a digital decision support tool that balances high predictive accuracy with clinical interpretability.

## 2 Materials and methods

### 2.1 Study design and ethical considerations

**FIGURE 1:**
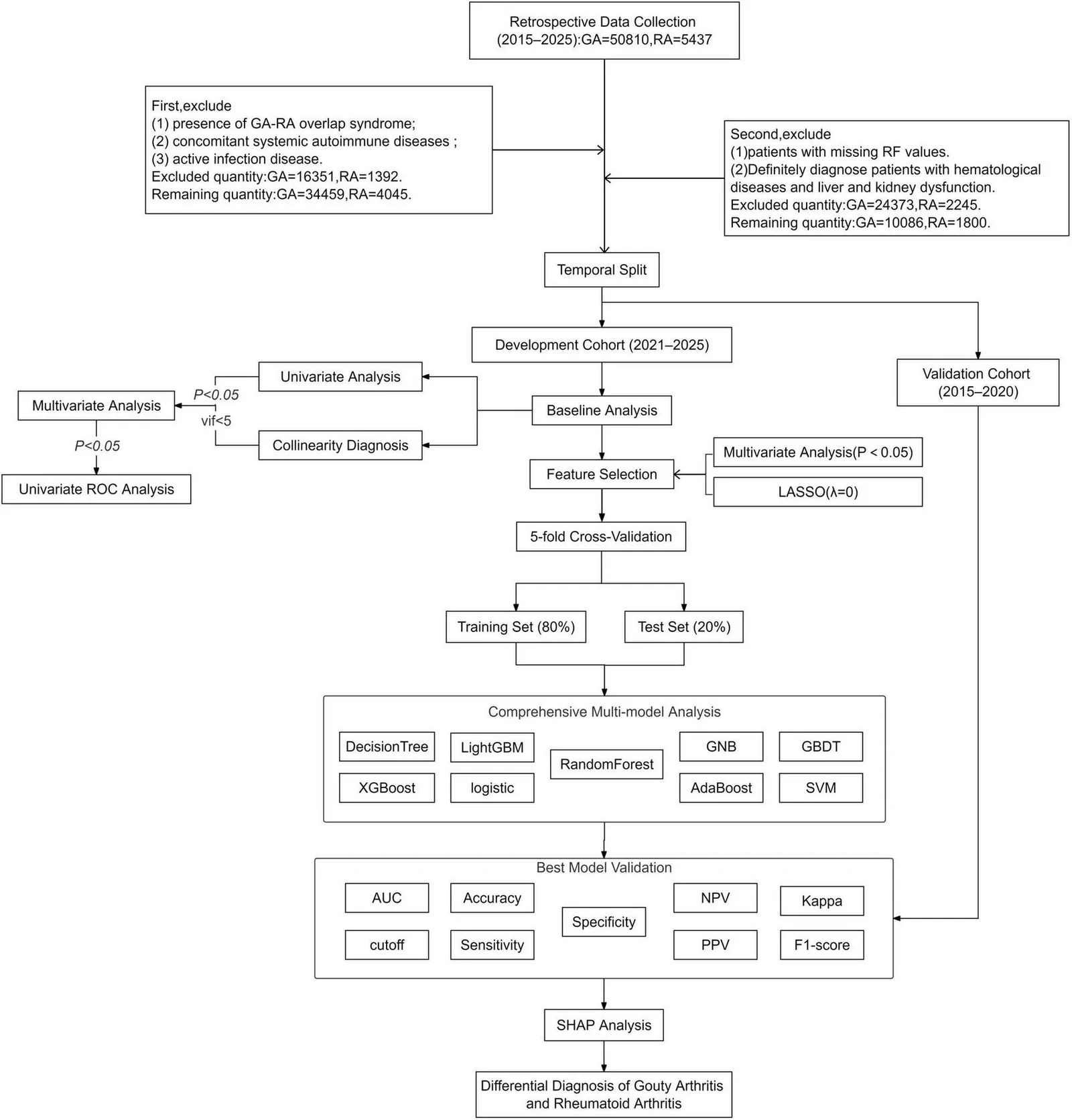
Idea design flow chart.

This single-center, retrospective observational study was conducted at The Third Affiliated Hospital of Guangzhou University of Chinese Medicine. The study aimed to develop and validate a machine learning (ML) model based on routine laboratory indices for the differential diagnosis of gouty arthritis and rheumatoid arthritis. the study protocol adhered strictly to the principles of the Declaration of Helsinki and was approved by the Institutional Ethics Committee of The Third Affiliated Hospital of Guangzhou University of Chinese Medicine (Approval No. PJ-XS-20260325-009). Given the retrospective nature of the study and the anonymization of patient data, the requirement for informed consent was waived. The overall study workflow is illustrated in Figure 1.

### 2.2 Cohort definition and data partitioning

To ensure model robustness and temporal stability, a temporal validation strategy was employed. The study population was stratified into two distinct cohorts based on the time of admission:

(1) Development Cohort: Patients admitted between January 1, 2021, and August 31, 2025. Due to the superior quality of recent data and adherence to updated clinical recording standards, this cohort was designated for feature selection, model training, and internal testing.
(2) Historical Validation Cohort: An independent group of patients admitted from January 1, 2015, to December 31, 2020. This cohort served as a temporally independent testing set to evaluate model performance on real-world historical data.

### 2.3 Inclusion and exclusion criteria

Patients were included if they met the following criteria: (1) a confirmed diagnosis of GA according to the 2015 ACR/EULAR criteria (20) or RA per the 2010 ACR/EULAR criteria (21); and (2) availability of complete baseline demographic and laboratory data at the initial visit.

Exclusion criteria encompassed: (1) presence of GA-RA overlap syndrome; (2) concomitant systemic autoimmune diseases (e.g., systemic lupus erythematosus, Sjögren’s syndrome); (3) active infection, malignancy, or end-stage liver/renal disease; and (4) > 20% missing data in key variables.

### 2.4 Data collection and feature engineering

Baseline clinical characteristics and laboratory indicators were all extracted from the electronic medical record (EMR) system. A total of 57 variables were included, covering complete blood count (RBC, WBC, HGB, and PLT), inflammatory markers (CRP and ESR), biochemical indicators (UA, Urea, Crea, TG, CHOL, HDL, and LDL), as well as immunological markers (RF and ASO). To explore the interplay between metabolism and inflammation, composite ratios were calculated: NLR, PLR, LMR, CRP/ CHOL, UA/HDL, and CHOL/TG, among others. These indicators were included as independent variables in subsequent analyses.

### 2.5 Machine learning framework

All data preprocessing, feature selection, and model development were executed on the Beckman Coulter DxAI platform.^1^

#### 2.5.1 Data preprocessing and feature selection

Missing values were imputed using the Multivariate Imputation by Chained Equations (MICE) method. Data are missing completely at random, and the number of imputations and convergence diagnostics were set to 5. The specific missing proportions for each variable were: Development cohort: Proportion of missing values: TG, CHOL, HDL, LDL, CHOL/TG is 5.12%, Crea is 15.15%, UA is 13.29%, etc. Verify that there are no missing values in the queue. First, variables were screened using binary logistic univariate analysis (*P* < 0.05) and collinearity diagnostics (VIF < 5), followed by multivariate binary logistic regression (*P* < 0.05) and univariate ROC analysis. Second, the least absolute shrinkage and selection operator (Lasso) algorithm with 10-fold cross-validation was applied to determine the optimal penalty parameter (λ), thereby identifying the most predictive feature subset. The final variables were taken as the intersection of those selected by Lasso (λ = 0) and by multivariate binary logistic regression (*P* < 0.05), namely Sex, Crea, UA, CHOL/TG, ESR, and RF.

#### 2.5.2 Model construction and evaluation

A total of nine machine learning algorithms were established: XGBoost, logistic regression, LightGBM, RandomForest, AdaBoost, DecisionTree, GBDT, GNB, and SVM. The study cohort was randomly divided into a training set and an internal validation set at a ratio of 8:2. In the training set, automatic grid search combined with 5-fold cross-validation was used to optimize hyperparameters. Model performance was evaluated using AUC, cutoff value, accuracy, sensitivity, specificity, PPV, NPV, F1-score, and Kappa. The reliability and clinical utility of the predicted probabilities were assessed via calibration curves (Brier score) and decision curve analysis (DCA), respectively.

#### 2.5.3 Model interpretability

To enhance the clinical transparency and interpretability of the optimal model, the game-theoretic SHAP (SHapley Additive exPlanations) framework was employed. Feature contributions were quantified at both the global (feature importance) and local (individual decision) levels.

### 2.6 Statistical analysis

Statistical analyses were performed within the Python (v3.11.4) and R (v4.2.3) environments integrated into the platform. Continuous variables were expressed as median (interquartile range, IQR) or mean standard deviation (SD), and inter-group comparisons were conducted using the Mann-Whitney U test or Student’s *t*-test. Categorical variables were presented as frequencies (%), and comparisons were made using the χ^2^ test. A two-sided *P* < 0.05 was considered statistically significant.

## 3 Results

### 3.1 Baseline characteristics of the study population

A total of 9903 subjects were enrolled in the final analysis, comprising 1629 patients with Rheumatoid Arthritis (RA) and 8274 with gouty arthritis (GA). Detailed baseline characteristics are summarized in Table 1. Demographic analysis revealed distinct inter-group disparities. The RA cohort was significantly female (76.12%). In stark contrast, the GA cohort was male-dominated (70.93%). Both age and sex distributions differed significantly between the two groups (*P* < 0.001). Regarding laboratory parameters, patients with GA exhibited significantly elevated levels of metabolic and hematological markers, specifically serum uric acid (UA) and creatinine compared to the RA group (all *P* < 0.001). Conversely, the RA group was characterized by a more pronounced inflammatory profile, with significantly higher levels of Erythrocyte sedimentation rate (ESR), and rheumatoid factor (RF). With the exception of NLR/PLR (*P* = 0.346), CO_2_ (*P* = 0.244), Urea (*P* = 0.35), PLT (*P* = 0.414), MCV (*P* = 0.143), HDL (*P* = 0.058) and NEUT (*P* = 0.86) all analyzed variables successfully discriminated between GA and RA patients with statistical significance. Additional details are provided in Supplementary Table 1.

**TABLE 1:** Demographic and clinical characteristics of the study population stratified by disease type and study cohort.

Variables	All (*N* = 9903)	RA (*n* = 1629)	GA (*n* = 8274)	*P*-value
Demographics				
Male, *n* (%)	6258(63.193)	389(23.880)	5869(70.933)	< 0.001
Female, *n* (%)	3645(36.807)	1240(76.120)	2405(29.067)	
Laboratory findings				
UA, median (IQR)	391.000[319.000, 484.000]	347.000[269.000, 391.000]	391.000[333.000, 512.000]	< 0.001
RF, median (IQR)	6.300[3.900, 12.300]	22.500[7.400, 69.400]	5.700[3.700, 9.400]	< 0.001
ESR, median (IQR)	15.000[6.000, 33.000]	21.300[8.600, 52.000]	13.000[5.700, 29.000]	< 0.001
Crea, median (IQR)	78.000[67.000, 88.000]	66.500[56.000, 78.000]	78.000[71.000, 89.410]	< 0.001
Combined ratios				
CHOL/TG, median (IQR)	2.647[1.938, 3.519]	2.774[2.642, 4.023]	2.586[1.865, 3.428]	< 0.001

### 3.2 Univariate evaluation, multicollinearity diagnosis, multivariable logistic regression analysis and univariate ROC analysis

**FIGURE 2:**
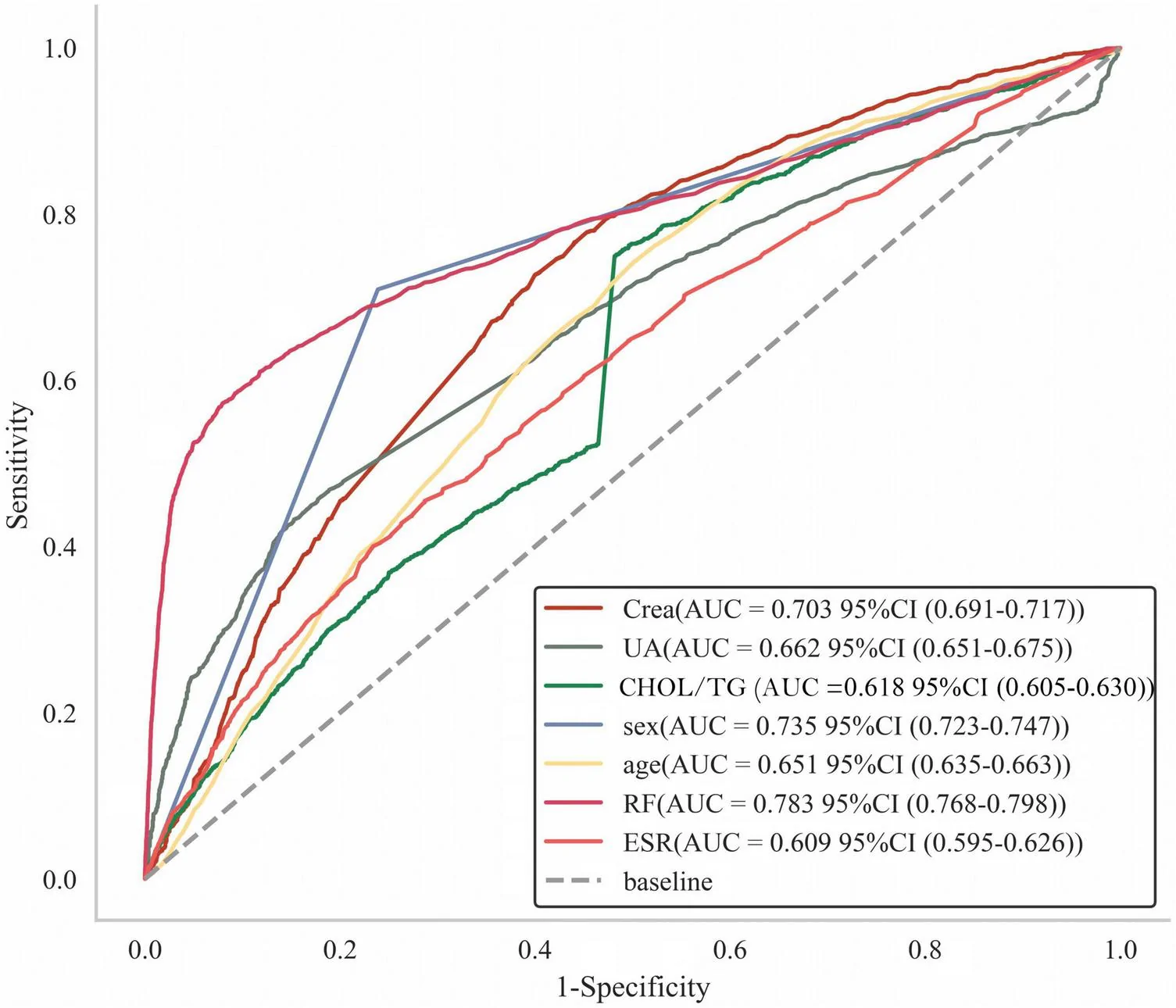
Single-factor ROC analysis.

Initial screening via univariate analysis identified candidate variables with *P* < 0.05 (Supplementary Table 2). Subsequently, collinearity diagnostics using the Variance Inflation Factor (VIF) were performed to eliminate redundant variables (VIF > 5) (Supplementary Table 3). Variables passing both screenings were subjected to multivariable analysis, which identified seven independent predictors with *P* < 0.05 (age, RF, ESR, CHOL/TG, UA, Crea, and sex; Table 2). Subsequent univariate ROC analysis revealed that RF demonstrated the highest individual diagnostic performance (AUC = 0.783, 95% CI: 0.768–0.798). However, other single markers exhibited suboptimal discriminative ability, proving insufficient for clinical differential diagnosis (Figure 2). These univariate assessments provided the foundational evidence for subsequent feature selection and multi-variable model construction.

**TABLE 2:** Multivariate logistic regression analysis.

Predictor	Estimate	SE	OR (95%CI)	*Z*	*p*
(Intercept)	0.906	0.207	2.473(1.646–3.709)	4.371	0.0
NEUT/MONO	0.001	0.005	1.001(0.994–1.011)	0.313	0.754
UA/Crea	0.017	0.016	1.017(0.986–1.049)	1.086	0.277
Crea	0.012	0.002	1.012(1.009–1.016)	7.073	0.0
UA	0.002	0.0	1.002(1.001–1.002)	6.529	0.0
Age	−0.01	0.002	0.990(0.985–0.995)	−4.167	0.0
CHOL/TG	−0.11	0.021	0.896(0.858–0.933)	−5.155	0.0
ESR	−0.003	0.001	0.997(0.994–0.999)	−2.636	0.008
ASO	0.0	0.0	1.000(0.999–1.001)	0.055	0.956
RF	−0.042	0.002	0.959(0.956–0.962)	−25.95	0.0
Sex (Male)	1.627	0.083	5.087(4.330–5.988)	19.676	0.0

Z, regression coefficient; SE, standard error; OR, odds ratio; CI, confidence interval.

### 3.3 LASSO-based feature selection

**FIGURE 3:**
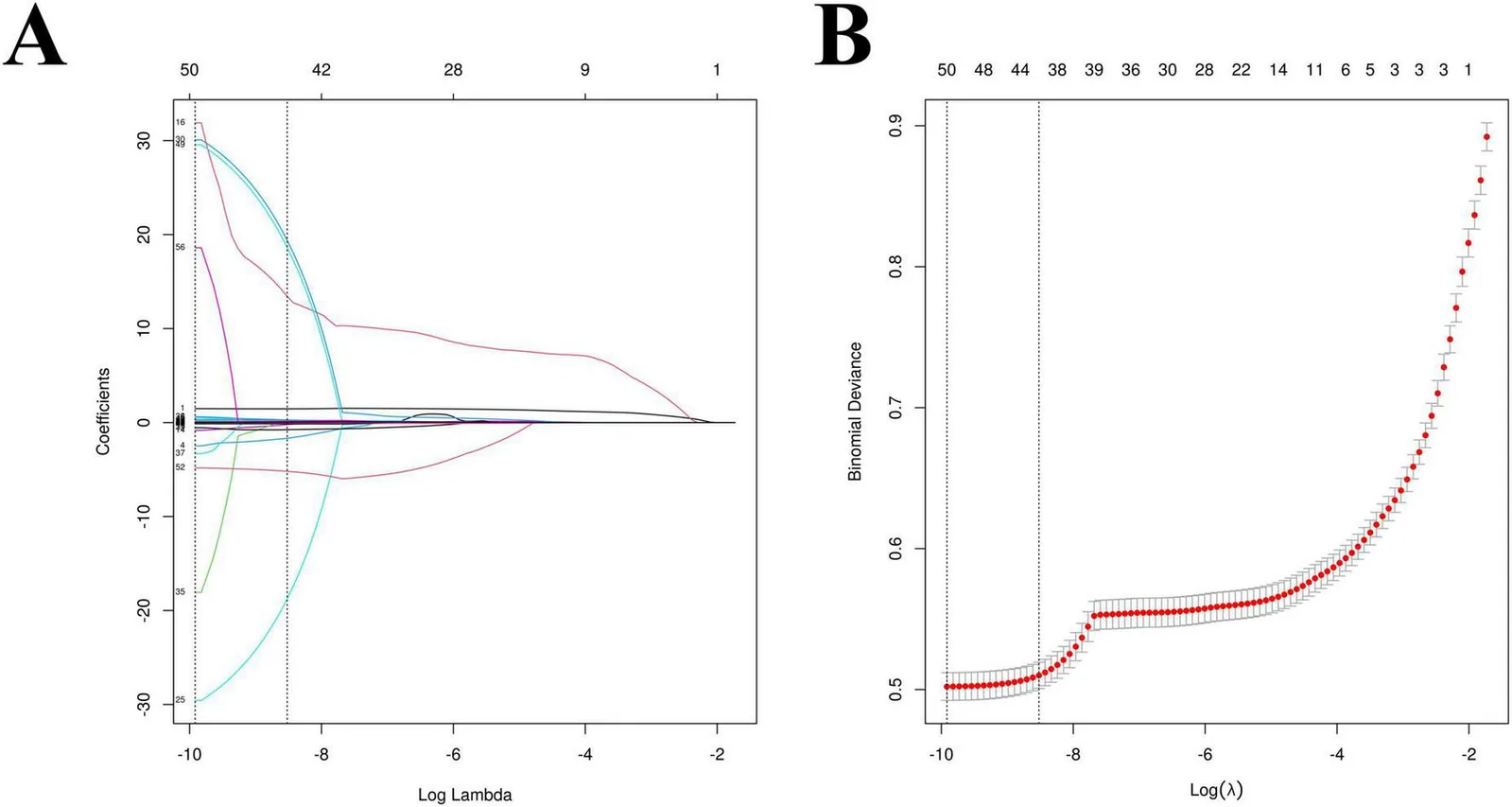
LASSO-based feature selection. **(A)** LASSO coefficient path diagram of clinical characteristics. **(B)** 10-fold cross-validation method is used to screen the optimal penalty coefficient (λ) in the LASSO model.

To optimize feature selection and mitigate overfitting, LASSO regression was employed (Figure 3). Figure 3A illustrates the coefficient profiles of the candidate variables. As the penalty parameter (λ) increased, the regression coefficients were progressively shrunk toward zero, thereby eliminating redundant features and retaining only the most informative predictors. Ten-fold cross-validation was performed to identify the optimal λ value corresponding to the minimum binomial deviance (Figure 3B). The λ value selected by the minimum criterion was 0. A total of 40 variables were retained in the final LASSO model. The coefficients under different selection criteria are presented in Supplementary Table 4. Since the LASSO method retained an excessive number of variables, we further applied multivariate analysis (*P* < 0.05) and selected the intersecting variables, namely RF, ESR, CHOL/TG, UA, Crea, and sex.

### 3.4 Performance comparison of nine models

**FIGURE 4:**
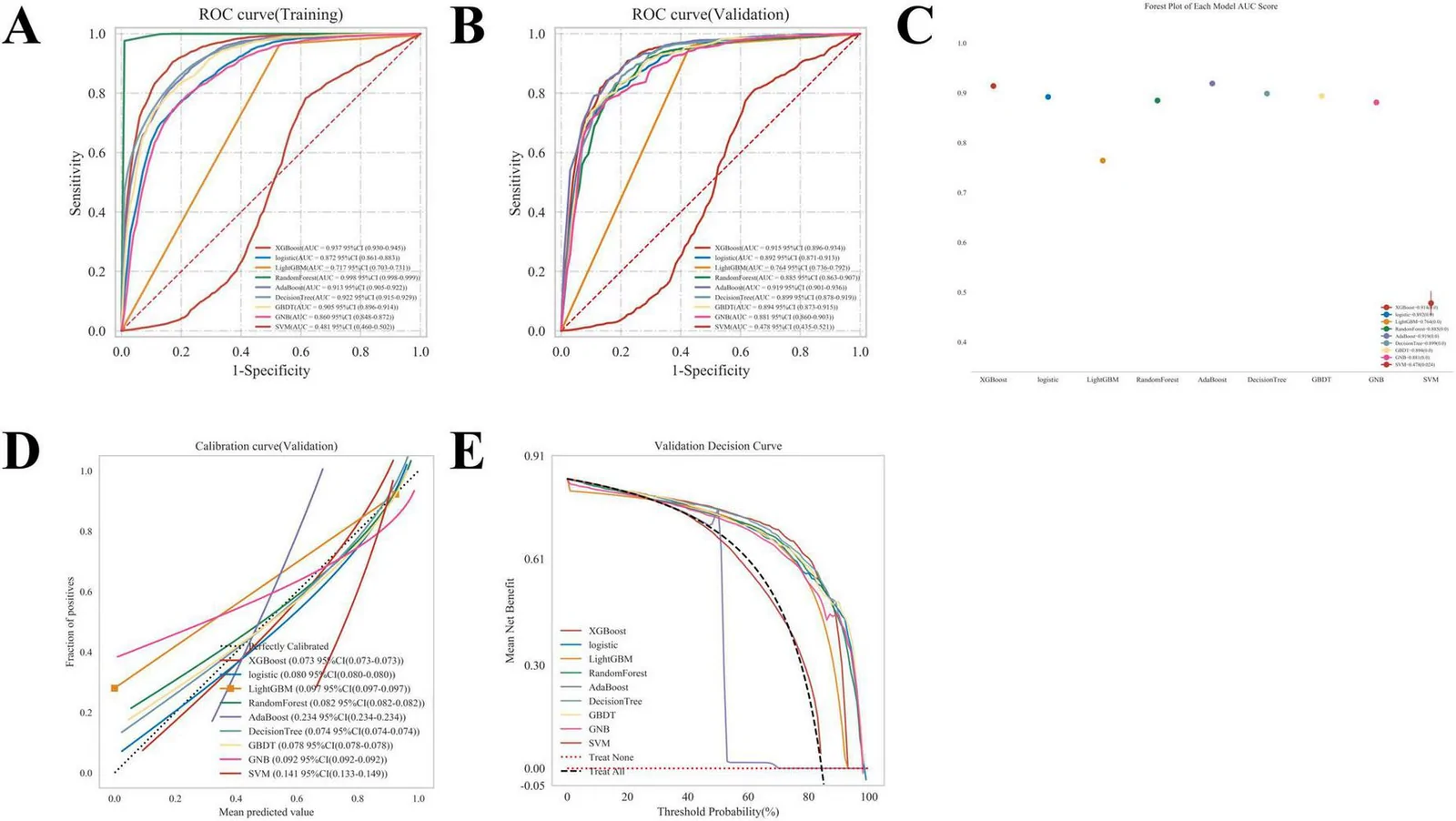
Performance comparison of nine models. **(A)** ROC analysis of the training set and **(B)** internal validation set. **(C)** AUC forest plot of the nine models. **(D)** Calibration curve of the validation set. **(E)** Decision curve analysis (DCA) of the validation set.

To determine the optimal diagnostic tool for distinguishing GA from RA, nine machine learning algorithms—XGBoost, Logistic Regression, LightGBM, Random Forest, AdaBoost, Decision Tree, GBDT, GNB, and SVM—were trained and evaluated using 5-fold cross-validation. Discriminative performance was quantified via the Area Under the ROC Curve (AUC). Comparative performance metrics for the training and validation sets are detailed in Supplementary Table 5 and Table 3, respectively. As illustrated in Figure 4 and Table 3, the AdaBoost model demonstrated superior robustness. RandomForest is very prone to overfitting, whereas AdaBoost might exhibit comparatively better stability. The AdaBoost model yielded an AUC of 0.913 (95% CI 0.905–0.922) in the training cohort (Figure 4A) and 0.919 (95% CI 0.901–0.936) in the internal validation cohort (Figure 4B). The DeLong test revealed that the AdaBoost model exhibited the highest AUC among all classifiers (Figure 4C). Based on the comprehensive evaluation of cutoff value, accuracy, sensitivity, specificity, PPV, NPV, F1-score, and Kappa, AdaBoost showed marginally superior performance relative to the other classifiers (Supplementary Table 5 and Table 3).

**TABLE 3:** Performance comparison of different machine learning models in the internal validation set.

Models	AUC (95%CI)	Cutoff (95%CI)	Accuracy (95%CI)	Sensitivity (95%CI)	Specificity (95%CI)	PPV (95%CI)	NPV (95%CI)	F1-score (95%CI)	Kappa (95%CI)
XGBoost	0.915 (0.896–0.934)	0.764(0.764–0.764)	0.878(0.878–0.878)	0.895(0.895–0.895)	0.788(0.788–0.788)	0.958(0.958–0.958)	0.583(0.583–0.583)	0.925(0.925–0.925)	0.598(0.598–0.598)
Logistic	0.892 (0.871–0.913)	0.825(0.825–0.825)	0.786(0.786–0.786)	0.774(0.774–0.774)	0.852(0.852–0.852)	0.966(0.966–0.966)	0.413(0.413–0.413)	0.859(0.859–0.859)	0.437(0.437–0.437)
LightGBM	0.764 (0.736–0.792)	0.925(0.925–0.925)	0.898(0.898–0.898)	0.959(0.959–0.959)	0.569(0.569–0.569)	0.923(0.923–0.923)	0.72(0.720–0.720)	0.94(0.940–0.940)	0.577(0.577–0.577)
RandomForest	0.885 (0.863–0.907)	0.64(0.640–0.640)	0.893(0.893–0.893)	0.935(0.935–0.935)	0.669(0.669–0.669)	0.938(0.938–0.938)	0.656(0.656–0.656)	0.936(0.936–0.936)	0.599(0.599–0.599)
AdaBoost	0.919 (0.901–0.936)	0.507(0.507–0.507)	0.867(0.867–0.867)	0.88(0.880–0.880)	0.794(0.794–0.794)	0.958(0.958–0.958)	0.553(0.553–0.553)	0.918(0.918–0.918)	0.573(0.573–0.573)
DecisionTree	0.899 (0.878–0.919)	0.837(0.837–0.837)	0.848(0.848–0.848)	0.856(0.856–0.856)	0.801(0.801–0.801)	0.958(0.958–0.958)	0.509(0.509–0.509)	0.904(0.904–0.904)	0.533(0.533–0.533)
GBDT	0.894 (0.873–0.915)	0.872(0.872–0.872)	0.804(0.804–0.804)	0.8(0.800–0.800)	0.826(0.826–0.826)	0.961(0.961–0.961)	0.435(0.435–0.435)	0.873(0.873–0.873)	0.458(0.458–0.458)
GNB	0.881 (0.860–0.903)	0.883(0.883–0.883)	0.799(0.799–0.799)	0.798(0.798–0.798)	0.807(0.807–0.807)	0.957(0.957–0.957)	0.426(0.426–0.426)	0.87(0.870–0.870)	0.443(0.443–0.443)
SVM	0.478 (0.435–0.521)	0.814(0.800–0.828)	0.731(0.710–0.752)	0.798(0.772–0.824)	0.367(0.362–0.372)	0.871(0.869–0.874)	0.256(0.230–0.282)	0.833(0.818–0.848)	0.141(0.115–0.168)

Since AUC solely assesses diagnostic accuracy without reflecting clinical utility, we further analyzed calibration curves and Decision Curve Analysis (DCA). The calibration curve (Figure 4D) confirmed that the AdaBoost calibration line most closely approximated the ideal diagonal, indicating high reliability in probability estimation. Furthermore, DCA (Figure 4E) indicated that the AdaBoost model provided the highest net benefit across a broad range of threshold probabilities, suggesting superior clinical applicability. Consequently, AdaBoost was designated as the optimal model for final evaluation.

### 3.5 Construction and evaluation of the optimal model (AdaBoost)

**FIGURE 5:**
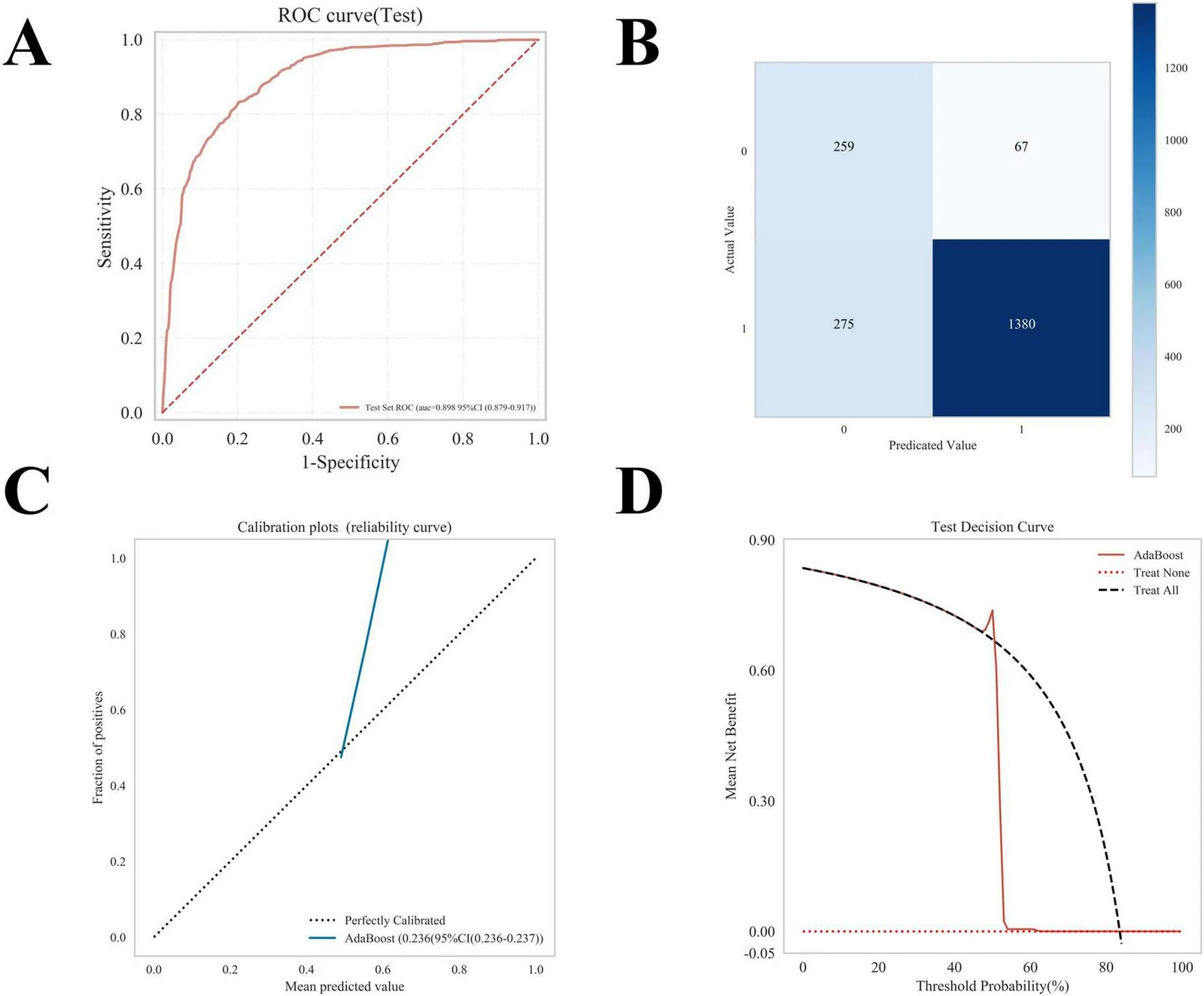
Construction and evaluation of the optimal model (AdaBoost). **(A)** Internal test cohort ROC analysis. **(B)** Confusion matrix plot. **(C)** Calibration curve. **(D)** Decision curve analysis (DCA).

Based on a comprehensive evaluation of performance metrics in the training and internal validation phases, AdaBoost was identified as the optimal classifier. To verify model robustness and rule out overfitting, we first assessed its performance in both sets. The AdaBoost model yielded an average AUC of 0.926 (95% CI: 0.915–0.936) in the training set (Supplementary Figure 1A), 0.897 (95% CI: 0.883–0.912) in the validation set (Supplementary Figure 1B) and AUC 0.898 (95%CI 0.879–0.917) in the test set (Figure 5A and Supplementary Table 6).

Classification efficacy was further assessed via the confusion matrix (Figure 5B), which showed the model correctly identified 1,380 GA and 259 RA patients, reflecting balanced sensitivity and specificity to effectively reduce misdiagnosis risk. Regarding reliability, the calibration curve (Figure 5C) showed high agreement between predicted and observed probabilities, with a Brier score of 0.236 (95% CI: 0.236–0.237). The fitted line adhered closely to the ideal diagonal, suggesting no significant risk of overestimation or underestimation. Additionally, DCA (Figure 5D) confirmed the model’s clinical utility and exhibits promising value for early disease diagnosis. Compared to the default strategies of “treat all” or “treat none,” the AdaBoost model offered significant net benefit across a wide range of threshold probabilities. This suggests that utilizing this model in clinical practice can significantly enhance decision-making accuracy for identifying GA patients without increasing unnecessary interventions.

### 3.6 Explanatory analysis of AdaBoost model based on SHAP

**FIGURE 6:**
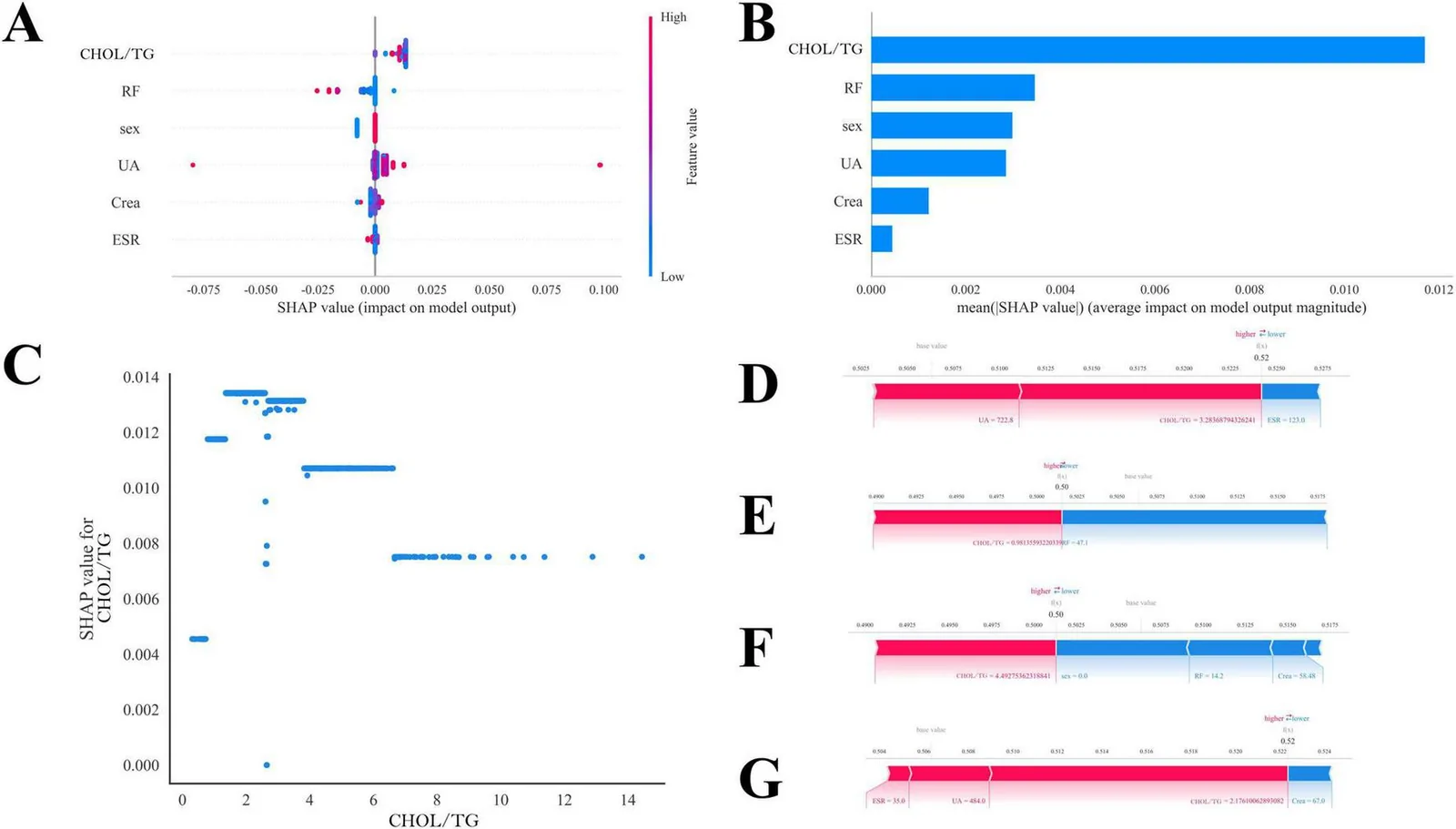
Explanatory analysis of AdaBoost model based on SHAP. **(A)** SHAP summary diagram; **(B)** feature importance ranking bar chart; **(C)** dependence plot for CHOL/TG; **(D–G)** SHAP force diagram for single patient prediction.

Although ensemble algorithms often achieve superior predictive performance, their inherent “black-box” nature may hinder clinical adoption. To bridge the gap between algorithmic performance and clinical interpretability, the SHAP framework was employed to explain the decision-making process of the optimal model. Figure 6A further illustrates how individual feature values impact model predictions. In this summary plot, each dot represents one sample, with red indicating higher feature values and blue indicating lower values. Figure 6B ranks the global feature importance according to the mean absolute SHAP values. The results identified CHOL/TG as the most influential predictor driving model classification, followed by RF, sex, UA, Crea, and ESR. Notably, CHOL/TG exhibited the highest importance, suggesting that lipid-related metabolic abnormalities may play a key role in distinguishing GA from RA. The distribution suggests that higher CHOL/TG values were associated with increased model output. Figure 6C presents the dependence plot for CHOL/TG, further indicating that variations in CHOL/TG can substantially influence the model output.

To demonstrate the model’s interpretability in real clinical scenarios, Figures 6D–G provide representative force plots for individual samples. These visualizations decompose the final predicted probability into the contribution of each feature, clearly showing how different variables jointly push the model output toward one class or the other. Overall, these findings indicate that the classification decision is mainly driven by CHOL/TG together with other clinical and laboratory indicators, while SHAP analysis enhances the transparency and clinical interpretability of the model.

### 3.7 Time-based internal validation

**FIGURE 7:**
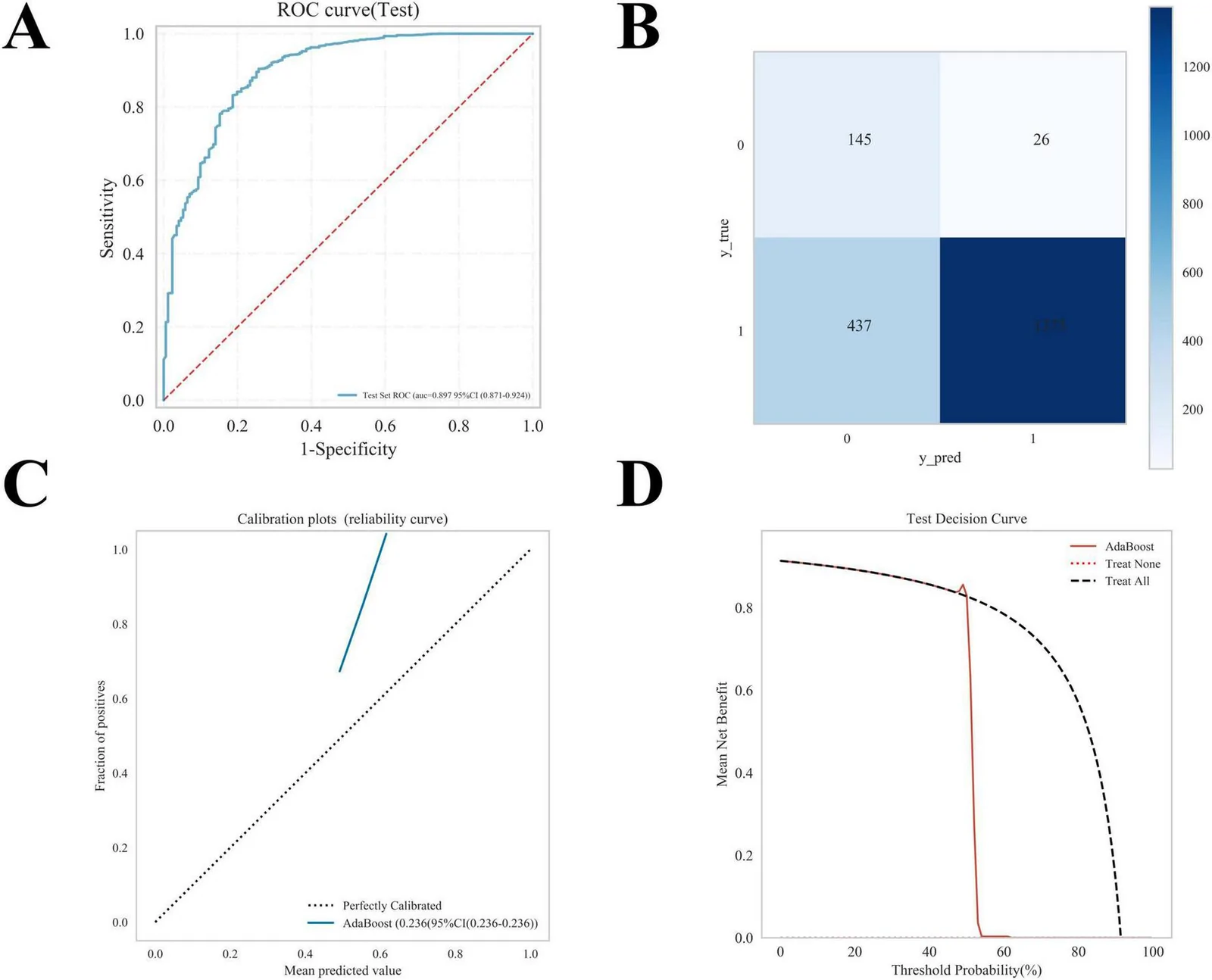
Performance evaluation of timing verification queue. **(A)** ROC analysis. **(B)** Confusion matrix plot. **(C)** Calibration curve. **(D)** Decision curve analysis (DCA).

To rigorously assess the temporal robustness of the AdaBoost model and its potential for real-world application, a Historical Validation Cohort comprising 1,983 patients admitted to the same institution between January 2015 and December 2020 was compiled to test generalization performance. Baseline characteristics are detailed in Supplementary Table 7. The model demonstrated exceptional generalization capabilities in this independent temporal cohort. As shown in Figure 7A, the AUC of 0.897 (95% CI: 0.871–0.924). This suggests that the identified key features retain high discriminative power over time. The confusion matrix (Figure 7B) correctly identifying 1,375 of 1,812 GA patients. Although sensitivity (0.759, Supplementary Table 8) saw a minor decline compared to the internal test set (0.834, Supplementary Table 6), this reflects a rational trade-off amidst the natural fluctuations of case composition in clinical practice.

Furthermore, the calibration curve (Figure 7C) yielded a Brier score of 0.236(95% CI: 0.236–0.236), indicating stable consistency between predicted risks and observed outcomes. DCA in the time-based internal validation (Figure 7D) continued to show high net benefit across relevant probability thresholds, confirming the model’s sustained value for clinical decision support in distinct patient populations.

## 4 Discussion

Rheumatoid arthritis (RA) and gouty arthritis (GA), the two most prevalent inflammatory arthropathies, frequently present with indistinguishable clinical manifestations—such as joint erythema, swelling, and warmth—during early or atypical stages, creating a significant diagnostic conundrum (22, 23). While synovial fluid crystal analysis remains the diagnostic gold standard, its routine implementation is hindered in primary care settings (24). Furthermore, reliance solely on serum uric acid or rheumatoid factor is often limited by insufficient specificity (25). Clinical evidence indicates that 30–50% of GA patients exhibit normouricemia or even hypouricemia during acute flares (3, 25)—a phenomenon termed “uric acid pseudonormalization”—which significantly confounds accurate diagnosis. To address this, we leveraged a large-scale retrospective cohort of 9,903 patients to pioneer an AdaBoost-based differential diagnostic model. Utilizing only routine hematological parameters, the model achieved high-precision discrimination, AUC0.898 in the internal validation set. Moreover, Decision Curve Analysis (DCA) demonstrated substantial net clinical benefit across a broad threshold probability range of 0.1–0.8. This suggests that our model functions not merely as a statistical classifier but as a pragmatic ancillary diagnostic tool adaptable to varying clinical risk preferences.

Importantly, the biological plausibility of our routine-laboratory-based model is supported by evidence suggesting that hematological inflammatory markers may reflect, at least in part, the systemic inflammatory burden of rheumatoid arthritis. CRP and ESR are conventional acute-phase reactants widely used in the assessment of inflammatory activity in RA (26), although their limitations in fully capturing synovial inflammation have also been recognized. Neutrophils, monocytes, lymphocytes, and platelets are involved in innate and adaptive immune responses, immune-cell recruitment, and inflammatory amplification in RA (27, 28). Accordingly, complete blood count-derived indices, including the neutrophil-to-lymphocyte ratio, monocyte-to-lymphocyte ratio, lymphocyte-to-monocyte ratio, platelet-to-lymphocyte ratio, and systemic immune-inflammatory index (29–31), have been increasingly investigated as accessible surrogate markers of systemic inflammation and disease activity. Notably, Atik et al. (32) reported that RA patients with lung involvement showed higher CRP, ESR, neutrophil counts, NLR, MLR, and SII, together with lower lymphocyte counts, suggesting that these hematological parameters may reflect not only articular inflammatory activity but also extra-articular pulmonary involvement. In the present study, RA patients also exhibited a more pronounced inflammatory profile, including higher CRP and ESR, and ESR remained an independent discriminator in multivariable analysis. Moreover, the monocyte–lymphocyte balance, represented by LMR in our feature set, and the CHOL/TG further contributed to disease classification. These findings indicate that the predictive performance of our model is not solely data-driven, but is biologically grounded in the distinct inflammatory and immunometabolic signatures that differentiate autoimmune RA from metabolically driven GA.

Through LASSO and multivariable logistic regression, we distilled 6 discriminative features from an initial pool of 57 variables. To mitigate the “black-box” opacity of machine learning, the SHAP framework was employed to visualize the contribution of each feature. As shown in Figure 6B, CHOL/TG, RF, and Sex emerged as the top three dominant predictors. This finding aligns closely with established clinical paradigms: Sex and UA reflect the epidemiological and metabolic underpinnings of GA, whereas RF serves as the serological hallmark of RA. However, beyond these traditional markers, our analysis illuminated the superior diagnostic efficacy of composite metabolic-inflammatory indices. Specifically, the CHOL/TG ranked prominently in feature importance. These composite ratios profoundly reflect the distinct pathophysiological mechanisms driving GA and RA. Notably, SHAP analysis identified the CHOL/TG ratio as the top-ranking composite feature, with lower ratios being significantly predictive of GA. GA is not merely an isolated articular disease but an articular manifestation of systemic metabolic syndrome (33). Its lipid profile is characterized by a “triglyceride-driven metabolic overload,” which exhibits high collinearity with insulin resistance and impaired renal uric acid excretion (34, 35). Consequently, the significantly elevated denominator (TG) in GA patients drives the CHOL/TG ratio to lower levels (36). In contrast, while RA patients also exhibit dyslipidemia, it is primarily driven by autoimmunity. During acute inflammation, elevated pro-inflammatory cytok*ines*(e.g.,*IL*−6,*TNF*−α) drastically downregulate hepatic cholesterol synthesis (37). This “high inflammation-low cholesterol” state, often referred to as the “lipid paradox,” leads to a rapid decline in the numerator (CHOL), resulting in a characteristic fluctuation in the CHOL/TG ratio (38). While single lipid parameters are susceptible to nutritional status or medication, the CHOL/TG ratio acts as a normalized metric that cancels out inter-individual variations in total lipid burden (39). Instead, it focuses on the “qualitative shift” in lipid metabolism: distinguishing the metabolic excess inherent to GA from the inflammatory consumption characteristic of RA.

The prominence of the UA/HDL ratio (UHR) in feature importance resonates with recent findings identifying UHR as a sensitive surrogate marker for metabolic risk (40, 41). Compared to solitary uric acid levels, UHR captures the systemic metabolic derangements specific to gout more comprehensively, transcending localized articular pathology (42). This elucidates why the ratio retains robust discriminatory power even during the acute phase, where “uric acid pseudonormalization” might otherwise obscure the diagnosis. Pathophysiologically, GA is intrinsically a metabolic disorder, frequently comorbid with metabolic syndrome components such as dyslipidemia and insulin resistance (43). Hyperuricemia is often accompanied by a specific atherogenic lipid profile, characterized by elevated triglycerides and reduced high-density lipoprotein cholesterol (HDL-C) (44, 45). In contrast, while RA patients may exhibit lipid alterations, these are primarily driven by autoimmune responses rather than systemic metabolic syndrome (14). Consequently, the UA/HDL ratio may reflect an underlying metabolic-immune interaction pattern that warrants further mechanistic investigation. By using HDL as the denominator, this composite index amplifies the “high metabolic burden” signal specific to GA (39), effectively distinguishing RA patients with incidental hyperuricemia from GA patients with a classic metabolic phenotype (46).

C-reactive protein (CRP) and erythrocyte sedimentation rate (ESR) are both elevated in acute phases of GA and RA. However, their kinetics differ: in GA, CRP often rises rapidly in response to MSU crystal-induced NLRP3 inflammasome activation, whereas in RA, CRP elevation is driven by persistent IL-6 signaling from synovial inflammation. The CRP/CHOL ratio, conversely, reveals the “lipid paradox” phenomenon unique to RA (37). Previous studies have established that during acute RA flares, intense systemic inflammation (elevated CRP) inhibits hepatic lipid synthesis and enhances lipid uptake by the reticuloendothelial system (47, 48), paradoxically suppressing total cholesterol levels. In GA patients, although CRP levels also rise during flares, the underlying metabolic background typically maintains higher lipid levels, resulting in less drastic ratio fluctuations (49).

Among the selected cell-count-derived indices, the Lymphocyte-to-Monocyte Ratio (LMR) may reflect a fundamental divergence in the immunological landscape: the balance between innate and adaptive immunity (29). GA is a classic autoinflammatory disease primarily driven by the innate immune system (43). The deposition of MSU crystals activates the NLRP3 inflammasome in monocytes and macrophages, leading to robust monocyte recruitment to inflamed joints (50). This innate-dominated process tends to increase the proportion of monocytes relative to lymphocytes (51). Conversely, RA is a prototypical autoimmune disease driven by adaptive immunity, characterized by the activation of T and B lymphocytes and the production of autoantibodies (21). Moreover, severe systemic inflammation in RA often leads to lymphopenia due to lymphocyte apoptosis or tissue sequestration. Therefore, the LMR finding generates the hypothesis that GA and RA may differ in their innate-adaptive immune balance, but this requires direct immunological validation (52)—providing a solid biological basis for its high importance in SHAP analysis. We performed SHAP explanation analysis on four patients and observed that the CHOL/TG ratio contributed the most to the differential diagnosis between GA and RA, suggesting its paramount importance in disease discrimination (Figures 6D–G).

In this study, we benchmarked nine distinct machine learning algorithms, among which AdaBoost demonstrated superior overall discriminative performance and robustness. Unlike traditional Logistic Regression, AdaBoost, as a boosting ensemble algorithm, does not rely on linear assumptions. This enables it to effectively capture complex nonlinear interactions among routine hematological indices (53). This capability underscores its superiority over linear models in distinguishing GA from RA, conditions that share overlapping inflammatory profiles. AdaBoost leverages an iterative reweighting mechanism. During training, it progressively assigns higher weights to misclassified samples—typically “borderline cases” with ambiguous clinical features—forcing the model to focus on the “gray zone” patients that are hardest to discriminate. This mechanism significantly enhances specificity while maintaining high sensitivity, achieving an optimal balance in diagnostic efficacy (54).

Furthermore, given the highly multidimensional interactivity of the included laboratory ratios, AdaBoost aggregates weak classifiers to precisely capture subtle yet critical nonlinear associations, such as the interplay between “inflammatory levels and metabolic load,” thereby reducing misdiagnosis risks caused by feature ambiguity. Decision Curve Analysis (DCA) and calibration curves further corroborated the clinical superiority of AdaBoost. It provided the highest net benefit across a broad range of threshold probabilities (55), and the high concordance between predicted probabilities and observed frequencies confirms that the model provides clinicians with an accurate and reliable ancillary decision-support tool.

In this study, the integration of machine learning with SHAP analysis enables clinicians to obtain not only a diagnostic probability but also a visual explanation of the indices that drive the decision for a specific patient, thereby greatly enhancing clinical trust. Compared with costly dual-energy CT or invasive arthrocentesis, our model relies solely on complete blood counts and readily available biochemical markers readily available in primary care. This confers high clinical utility, making it particularly suitable for rapid triage in emergency departments or community hospitals. However, several limitations must be acknowledged. First, the study is inherently retrospective. Although it spans a decade of clinical data, it is susceptible to selection bias. Second, the Brier scores corresponding to Figures 5C, 7C were 0.236. The close values indicate good consistency in the model’s predictive performance across different validation scenarios; however, the overall high scores indicate suboptimal probabilistic calibration, with considerable deviation in risk estimates for disease diagnosis. Third, despite the large sample size derived from multiple campuses of a single institution, future external verification across multi-center cohorts is requisite to confirm model robustness. While age and other factors show significant effects in this single-center cohort, population heterogeneity may compromise the model’s performance. Fourth, potential limitations include the possible presence of undiagnosed hematological disorders and renal impairment among the study population. Hematological disorders might influence routine blood parameters, whereas renal impairment could affect uric acid excretion. Furthermore, it is possible that some patients with gouty arthritis had already undergone medical therapy. Additionally, incorporating patient medication history, such as the use of urate-lowering therapies or corticosteroids, could further refine the model’s precision.

## 5 Conclusion

In this study, we successfully developed and validated an AdaBoost-based predictive model that leverages routine hematological indices to precisely differentiate gouty arthritis (GA) from Rheumatoid Arthritis (RA). Beyond superior performance in internal testing, the model demonstrated exceptional generalization and robustness in an independent historical temporal cohort, proving its resilience against the natural fluctuations inherent to dynamic clinical environments. Using SHAP interpretability analysis, we elucidated the critical pathophysiological significance of composite ratios, such asCHOL/TG, which act as “metabolic hub.” These metrics clarify the distinct interaction patterns between metabolic dysregulation and systemic inflammation in the two diseases. In conclusion, our model provides a non-invasive, cost-effective, and highly accurate auxiliary diagnostic tool. By incorporating RF, ESR, CHOL/TG, UA, Crea, and sex, it is expected to optimize early triage strategies and holds broad application prospects in resource-limited primary care settings.

## Data Availability

The original contributions presented in the study are included in the article/Supplementary material, further inquiries can be directed to the corresponding author.

## Footnotes

1
https://www.xsmartanalysis.com/beckman/login/

## References

[1] Punjwani S Jani C Liu W Kakoullis L Salciccioli I Al Omari O et al. Burden of gout among different WHO regions, 1990-2019: estimates from the global burden of disease study. *Sci Rep* (2024) 14:15953. 10.1038/s41598-024-61616-z 38987583 PMC11236997

[2] Wu CL Chang CC Kor CT Yang TH Chiu PF Tarng DC et al. Hydroxychloroquine use and risk of CKD in patients with rheumatoid arthritis. *Clin J Am Soc Nephrol.* (2018) 13:702–9. 10.2215/cjn.11781017 29661770 PMC5969483

[3] Roddy E Choi HK. Epidemiology of gout. *Rheum Dis Clin North Am.* (2014) 40:155–75. 10.1016/j.rdc.2014.01.001 24703341 PMC4119792

[4] Sayah A English JC. Rheumatoid arthritis: a review of the cutaneous manifestations. *J Am Acad Dermatol* (2005) 53:191–209. 10.1016/j.jaad.2004.07.023 16021111

[5] Sanghavi N Korem S Dey S Wasserman A Ash J. Dual-energy computed tomography (DECT) resolves the diagnostic dilemma in an atypically presenting case of gout. *Cureus.* (2023) 15:e38247. 10.7759/cureus.38247 37252479 PMC10225115

[6] Shiu WHL Cheng HMJ Chan YT Chu CY Kan WK. Gouty tophus: unusual case of nasal lump. *Radiol Case Rep.* (2021) 16:2904–7. 10.1016/j.radcr.2021.06.094 34401022 PMC8350013

[7] Dalbeth N Gosling AL Gaffo A Abhishek A. Gout. *Lancet.* (2021) 397:1843–55. 10.1016/s0140-6736(21)00569-9 33798500

[8] Yokose C Dalbeth N Wei J Nicolaou S Simeone FJ Baumgartner S et al. Radiologic evidence of symmetric and polyarticular monosodium urate crystal deposition in gout - a cluster pattern analysis of dual-energy CT. *Semin Arthritis Rheum* (2020) 50:54–8. 10.1016/j.semarthrit.2019.07.002 31371194 PMC6954342

[9] Smolen JS Aletaha D McInnes IB. Rheumatoid arthritis. *Lancet.* (2016) 388:2023–38. 10.1016/s0140-6736(16)30173-8 27156434

[10] Horino T Kashio T Inotani S Terada Y. Overhanging edge bone erosion in chronic tophaceous gout. *Intern Med.* (2020) 59:2335–6. 10.2169/internalmedicine.4648-20 32536646 PMC7578596

[11] van der Linden MP le Cessie S Raza K van der Woude D Knevel R Huizinga TW. Long-term impact of delay in assessment of patients with early arthritis. (2010) 62:3537–46. 10.1002/art.27692 20722031

[12] Nunes EA Rosseti AG Jr. Ribeiro DS Santiago M. Gout initially mimicking rheumatoid arthritis and later cervical spine involvement. *Case Rep Rheumatol.* (2014) 2014:357826. 10.1155/2014/357826 25574418 PMC4276112

[13] Collins K Aspey H Todd A Saravanan V Rynne M Kelly C. Methotrexate pneumonitis precipitated by switching from oral to parenteral administration. *Rheumatology.* (2008) 47:109–10. 10.1093/rheumatology/kem230 18042567

[14] Tian X Wang Q Jiang N Zhao Y Huang C Liu Y et al. Chinese guidelines for the diagnosis and treatment of rheumatoid arthritis: 2024 update. *Rheumatol Immunol Res.* (2024) 5:189–208. 10.1515/rir-2024-0028 39802551 PMC11720473

[15] Gabriel SE Michaud K. Epidemiological studies in incidence, prevalence, mortality, and comorbidity of the rheumatic diseases. *Arthritis Res Ther.* (2009) 11:229. 10.1186/ar2669 19519924 PMC2714099

[16] Richette P Doherty M Pascual E Barskova V Becce F Castaneda J et al. 2018 updated European league against rheumatism evidence-based recommendations for the diagnosis of gout. *Ann Rheum Dis.* (2020) 79:31–8. 10.1136/annrheumdis-2019-215315 31167758

[17] Pisaniello HL Lester S Gonzalez-Chica D Stocks N Longo M Sharplin GR et al. Gout prevalence and predictors of urate-lowering therapy use: results from a population-based study. *Arthritis Res Ther.* (2018) 20:143. 10.1186/s13075-018-1633-9 29996922 PMC6042461

[18] Hügle M Omoumi P van Laar JM Boedecker J Hügle T. Applied machine learning and artificial intelligence in rheumatology. *Rheumatol Adv Pract.* (2020) 4:rkaa005. 10.1093/rap/rkaa005 32296743 PMC7151725

[19] Gan M Peng Y Ying Y Zhang K Chen Y. Machine learning differentiation of rheumatoid arthritis-sjögren’s syndrome overlap from sjögren’s syndrome with polyarthritis. *Front Immunol.* (2025) 16:1614631. 10.3389/fimmu.2025.1614631 40698089 PMC12280366

[20] Neogi T Jansen TL Dalbeth N Fransen J Schumacher HR Berendsen D et al. 2015 Gout classification criteria: an american college of rheumatology/european league against rheumatism collaborative initiative. *Arthritis Rheumatol.* (2015) 67:2557–68. 10.1002/art.39254 26352873 PMC4566153

[21] Le Loët X Nicolau J Boumier P Daragon A Mejjad O Pouplin S et al. Validation of the 2010-ACR/EULAR -classification criteria using newly EULAR-defined erosion for rheumatoid arthritis on the very early arthritis community-based (VERA) cohort. *Joint Bone Spine.* (2015) 82:38–41. 10.1016/j.jbspin.2014.03.008 25304188

[22] Kiefer D Erkenberg J Braun J. Similarities and differences between gouty arthritis and rheumatoid arthritis—an interesting case with a short look into the literature. *Exo Musculoskeletal Dis.* (2023) 1:11–9. 10.37349/emd.2023.00003

[23] Xue SW Luo YK Zhao YR Jiao ZY. Musculoskeletal ultrasound in the differential diagnosis of gouty arthritis and rheumatoid arthritis. *Pak J Med Sci.* (2020) 36:977–81. 10.12669/pjms.36.5.2716 32704274 PMC7372673

[24] Lee KH Choi ST Lee SK Lee JH Yoon BY. Application of a novel diagnostic rule in the differential diagnosis between acute gouty arthritis and septic arthritis. *J Korean Med Sci.* (2015) 30:700–4. 10.3346/jkms.2015.30.6.700 26028920 PMC4444468

[25] Zhang W Doherty M Pascual E Bardin T Barskova V Conaghan P et al. EULAR evidence based recommendations for gout. *Ann Rheum Dis.* (2006) 65:1301–11. 10.1136/ard.2006.055251 16707533 PMC1798330

[26] Orr CK Najm A Young F McGarry T Biniecka M Fearon U et al. The utility and limitations of CRP, ESR and DAS28-CRP in appraising disease activity in rheumatoid arthritis.. *Front Med .* (2018) 5:185. 10.3389/fmed.2018.00185 30123796 PMC6085449

[27] Boilard E Larabee K Shnayder R Jacobs K Farndale RW Ware J et al. Platelets participate in synovitis via Cox-1-dependent synthesis of prostacyclin independently of microparticle generation. *J Immunol.* (2011) 186:4361–6. 10.4049/jimmunol.1002857 21357261 PMC3208424

[28] Boilard E Nigrovic PA Larabee K Watts GF Coblyn JS Weinblatt ME et al. Platelets amplify inflammation in arthritis via collagen-dependent microparticle production. *Science.* (2010) 327:580–3. 10.1126/science.1181928 20110505 PMC2927861

[29] Targońska-Stępniak B Zwolak R Piotrowski M Grzechnik K Majdan M. The relationship between hematological markers of systemic inflammation (neutrophil-to-lymphocyte, platelet-to-lymphocyte, lymphocyte-to-monocyte ratios) and ultrasound disease activity parameters in patients with rheumatoid arthritis. *J Clin Med.* (2020) 9:2760. 10.3390/jcm9092760 32858869 PMC7564422

[30] Du J Chen S Shi J Zhu X Ying H Zhang Y et al. The association between the lymphocyte-monocyte ratio and disease activity in rheumatoid arthritis. *Clin Rheumatol.* (2017) 36:2689–95. 10.1007/s10067-017-3815-2 28913574

[31] Okutan İ Aci R Keskin Â Bilgin M Kızılet H. New inflammatory markers associated with disease activity in rheumatoid arthritis: pan-immune-inflammation value, systemic immune-inflammation index, and systemic inflammation response index. *Reumatologia.* (2024) 62:439–46. 10.5114/reum/196066 39866302 PMC11758101

[32] Atik S Apalan D Atik İ. Diagnostic contribution of hematological parameters in patients with lung involvement in rheumatoid arthritis. *Health Sci Institute.* (2024) 9:56–60. 10.51754/cusbed.1423583

[33] Zheng B Sun J Luo H Yang L Li Q Zhang L et al. Testosterone protects mice against zika virus infection and suppresses the inflammatory response in the brain. *IScience.* (2022) 25:105300. 10.1016/j.isci.2022.105300 36304103 PMC9593801

[34] Dehlin M Jacobsson L Roddy E. Global epidemiology of gout: prevalence, incidence, treatment patterns and risk factors. *Nat Rev Rheumatol.* (2020) 16:380–90. 10.1038/s41584-020-0441-1 32541923

[35] Rios Santos KK Chohan F Simo RE Chambi-Torres JB Michel G. Severe tophaceous polyarticular gout: a case report and review of literature. *Cureus.* (2025) 17:e79045. 10.7759/cureus.79045 40099075 PMC11912807

[36] Choi IA Kim JH Lee YJ Kang EH Ha YJ Shin K et al. Performance of the 2015 American College of rheumatology/european league against rheumatism classification criteria for gout in korean patients with acute arthritis. *J Korean Med Sci.* (2019) 34:e155. 10.3346/jkms.2019.34.e155 31172694 PMC6556442

[37] Myasoedova E Crowson CS Kremers HM Roger VL Fitz-Gibbon PD Therneau TM et al. Lipid paradox in rheumatoid arthritis: the impact of serum lipid measures and systemic inflammation on the risk of cardiovascular disease. *Ann Rheum Dis.* (2011) 70:482–7. 10.1136/ard.2010.135871 21216812 PMC3058921

[38] Shi Y Zhou M Chang C Jiang P Wei K Zhao J et al. Advancing precision rheumatology: applications of machine learning for rheumatoid arthritis management. *Front Immunol.* (2024) 15:1409555. 10.3389/fimmu.2024.1409555 38915408 PMC11194317

[39] Singh JA Cleveland J. Epidemiology of cardiac or orthopedic procedures in gout versus rheumatoid arthritis: a national time-trends study. *Ther Adv Musculoskelet Dis.* (2021) 13:1759720x20973916. 10.1177/1759720x20973916 33737964 PMC7934033

[40] Posadas-Sánchez R Fuentevilla-Álvarez G Vargas-Alarcón G Cardoso-Saldaña GC. Is UA/HDL-C a reliable surrogate marker for fatty liver? a comparative evaluation with metabolic scores in a mexican population: the genetics of atherosclerotic disease study. *Diagnostics.* (2025) 15: 1419. 10.3390/diagnostics15111419 40506991 PMC12154243

[41] Yazdi F Baghaei MH Baniasad A Naghibzadeh-Tahami A Najafipour H Gozashti MH. Investigating the relationship between serum uric acid to high-density lipoprotein ratio and metabolic syndrome. *Endocrinol Diabetes Metab.* (2022) 5:e00311. 10.1002/edm2.311 34705333 PMC8754234

[42] Kolahi Ahari R Mansoori A Sahranavard T Miri MS Feizi S Esmaily H et al. Serum uric acid to high-density lipoprotein ratio as a novel indicator of inflammation is correlated with the presence and severity of metabolic syndrome: a large-scale study. *Endocrinol Diabetes Metab.* (2023) 6:e446. 10.1002/edm2.446 37605374 PMC10638626

[43] Amatucci AJ Padnick-Silver L LaMoreaux B Bulbin DH. Comparison between early-onset and common gout: a systematic literature review. *Rheumatol Ther.* (2023) 10:809–23. 10.1007/s40744-023-00565-x 37335432 PMC10326179

[44] Huang Y Li Y Wu Z Liang Y He J. Exploring the associations and potential mediators between lipid biomarkers and the risk of developing gout: NHANES 2007-2018. *Lipids Health Dis.* (2024) 23:363. 10.1186/s12944-024-02346-z 39511685 PMC11542235

[45] Kvasnièka A Friedecký D Brumarová R Pavlíková M Pavelcová K Mašínová J et al. Alterations in lipidome profiles distinguish early-onset hyperuricemia, gout, and the effect of urate-lowering treatment. *Arthritis Res Ther.* (2023) 25:234. 10.1186/s13075-023-03204-6 38042879 PMC10693150

[46] Mirza T Heriansyah T Zufry H Muqsith M. Uric Acid-HDL Ratio as predictor of major cardiovascular events in very high cardiovascular risk patients treated with high-intensity statin therapy. *South East Eur J Public Health.* (2024) 25:1078–83. 10.70135/seejph.vi.2280

[47] Behl T Kaur I Sehgal A Zengin G Brisc C Brisc MC et al. The lipid paradox as a metabolic checkpoint and its therapeutic significance in ameliorating the associated cardiovascular risks in rheumatoid arthritis patients. *Int J Mol Sci.* (2020) 21:9505. 10.3390/ijms21249505 33327502 PMC7764917

[48] Pérez-Baos S Barrasa JI Gratal P Larrañaga-Vera A Prieto-Potin I Herrero-Beaumont G et al. Tofacitinib restores the inhibition of reverse cholesterol transport induced by inflammation: understanding the lipid paradox associated with rheumatoid arthritis. *Br J Pharmacol.* (2017) 174:3018–31. 10.1111/bph.13932 28646516 PMC5573422

[49] Fernández-Ortiz AM Ortiz AM Pérez S Toledano E Abásolo L González-Gay MA et al. Effects of disease activity on lipoprotein levels in patients with early arthritis: can oxidized LDL cholesterol explain the lipid paradox theory? *Arthritis Res Ther.* (2020) 22:213. 10.1186/s13075-020-02307-8 32917272 PMC7488761

[50] Wang Z Zhao Y Phipps-Green A Liu-Bryan R Ceponis A Boyle DL et al. Differential DNA methylation of networked signaling, transcriptional, innate and adaptive immunity, and osteoclastogenesis genes and pathways in gout. *Arthritis Rheumatol.* (2020) 72:802–14. 10.1002/art.41173 31738005 PMC7323903

[51] Huang Q Huang Y Guo X Chen J Zhong Z Liu Y et al. The diagnostic value of synovial fluid lymphocytes in gout patients. *Dis Markers.* (2021) 2021:4385611. 10.1155/2021/4385611 34567284 PMC8460379

[52] Taha SI Samaan SF Ibrahim RA Moustafa NM El-Sehsah EM Youssef MK. Can complete blood count picture tell us more about the activity of rheumatological diseases? *Clin Med Insights Arthritis Musculoskelet Disord.* (2022) 15:11795441221089182. 10.1177/11795441221089182 35481333 PMC9036329

[53] Dudek G Sakowski S Brzezińska O Sarnik J Budlewski T Dragan G et al. Machine learning-based prediction of rheumatoid arthritis with development of ACPA autoantibodies in the presence of non-HLa genes polymorphisms. *PLoS One.* (2024) 19:e0300717. 10.1371/journal.pone.0300717 38517871 PMC10959370

[54] Freund Y Schapire RE. A decision-theoretic generalization of on-line learning and an application to boosting. *J Comput Syst Sci.* (1997) 55:119–39. 10.1006/jcss.1997.1504

[55] Vickers AJ Van Calster B Steyerberg EW. Net benefit approaches to the evaluation of prediction models, molecular markers, and diagnostic tests. *BMJ.* (2016) 352:i6. 10.1136/bmj.i6 26810254 PMC4724785

