# Supplementary material for: AdaBoost-based differential diagnosis of gouty arthritis and rheumatoid arthritis: model construction, temporal validation, and SHAP visualization
Source: Front Med (Lausanne). 2026 Aug 10;13:1855696. doi: 10.3389/fmed.2026.1855696 (PMC13475015; doi:10.3389/fmed.2026.1855696)
Supplement: Supplementary file 1 [file Data_Sheet_1.docx]

**Supplementary Material**

**Supplementary Table S1. Detailed Laboratory Indicators and Calculated Ratios (N=9903)**

| **Variables (median [IQR])** | **Overall (N=9903)** | **RA (n=1,629)** | **GA (n=8,274)** | ***P*-value** |
| --- | --- | --- | --- | --- |
| age ,median[IQR] | 53.000[40.000,65.000] | 61.000[51.000,70.000] | 52.000[38.000,64.000] | <0.001 |
| WBC ,median[IQR] | 7.080[5.880,8.560] | 6.730[5.560,8.280] | 7.140[5.940,8.610] | <0.001 |
| BASO ,median[IQR] | 0.030[0.020,0.050] | 0.030[0.020,0.040] | 0.030[0.020,0.050] | <0.001 |
| NEUT ,median[IQR] | 4.170[3.250,5.450] | 4.220[3.210,5.560] | 4.160[3.260,5.430] | 0.860 |
| MONO ,median[IQR] | 0.420[0.340,0.540] | 0.420[0.330,0.540] | 0.430[0.340,0.540] | 0.002 |
| LYMPH ,median[IQR] | 2.070[1.630,2.530] | 1.780[1.370,2.240] | 2.130[1.690,2.580] | <0.001 |
| EO ,median[IQR] | 0.011[0.006,0.020] | 0.008[0.004,0.015] | 0.012[0.006,0.021] | <0.001 |
| BASO rate ,median[IQR] | 0.500[0.300,0.700] | 0.400[0.200,0.600] | 0.500[0.300,0.700] | <0.001 |
| NEUT rate ,median[IQR] | 59.900[53.700,66.900] | 63.800[56.300,70.600] | 59.300[53.300,66.000] | <0.001 |
| MONO rate ,median[IQR] | 6.100[5.100,7.200] | 6.100[5.100,7.500] | 6.000[5.100,7.100] | 0.008 |
| LYMPH rate ,median[IQR] | 30.300[23.600,36.300] | 26.900[20.300,33.600] | 30.800[24.400,36.700] | <0.001 |
| EO rate ,median[IQR] | 0.160[0.090,0.260] | 0.130[0.070,0.210] | 0.170[0.100,0.270] | <0.001 |
| RBC ,median[IQR] | 4.740[4.300,5.140] | 4.290[3.890,4.680] | 4.820[4.410,5.180] | <0.001 |
| HGB ,median[IQR] | 139.000[126.000,150.000] | 124.000[110.000,135.000] | 142.000[130.000,152.000] | <0.001 |
| HCT ,median[IQR] | 0.420[0.389,0.450] | 0.380[0.340,0.410] | 0.430[0.398,0.450] | <0.001 |
| MCV ,median[IQR] | 89.588[86.283,92.545] | 89.796[85.253,93.596] | 89.555[86.397,92.409] | 0.143 |
| MCH ,median[IQR] | 29.783[28.511,30.833] | 29.589[27.681,30.990] | 29.810[28.628,30.814] | <0.001 |
| MCHC ,median[IQR] | 330.435[323.077,337.500] | 326.829[318.919,334.601] | 331.096[323.913,337.838] | <0.001 |
| RDWCV ,median[IQR] | 12.900[12.400,13.700] | 13.300[12.600,14.600] | 12.800[12.300,13.500] | <0.001 |
| PLT ,median[IQR] | 253.000[214.000,299.000] | 253.000[211.000,308.000] | 253.000[215.000,297.000] | 0.414 |
| RDWSD ,median[IQR] | 42.200[39.900,44.600] | 43.800[41.100,47.000] | 41.900[39.800,44.100] | <0.001 |
| LDL ,median[IQR] | 3.000[2.470,3.630] | 3.000[2.490,3.320] | 3.000[2.470,3.680] | <0.001 |
| CHOL ,median[IQR] | 5.300[4.550,5.970] | 5.300[4.550,5.630] | 5.300[4.550,6.100] | <0.001 |
| Urea ,median[IQR] | 5.300[4.400,6.400] | 5.300[4.300,6.400] | 5.300[4.400,6.400] | 0.350 |
| TG ,median[IQR] | 1.950[1.460,2.730] | 1.830[1.260,1.950] | 2.000[1.500,2.820] | <0.001 |
| CO2 ,median[IQR] | 25.400[23.400,27.200] | 25.300[23.300,27.100] | 25.400[23.400,27.200] | 0.244 |
| HDL ,median[IQR] | 1.400[1.180,1.690] | 1.400[1.250,1.650] | 1.400[1.170,1.700] | 0.058 |
| ASO ,median[IQR] | 29.000[15.000,61.000] | 24.000[13.000,48.000] | 32.000[15.000,63.900] | <0.001 |
| CRP ,median[IQR] | 3.100[2.000,11.400] | 5.300[2.000,16.000] | 2.700[2.000,9.500] | <0.001 |
| WBC/PLT ,median[IQR] | 0.028[0.023,0.034] | 0.027[0.022,0.033] | 0.028[0.023,0.034] | <0.001 |
| WBC/RBC ,median[IQR] | 1.499[1.234,1.839] | 1.577[1.295,1.973] | 1.483[1.221,1.815] | <0.001 |
| WBC/HGB ,median[IQR] | 0.052[0.042,0.064] | 0.055[0.045,0.069] | 0.051[0.042,0.062] | <0.001 |
| HGB/RBC ,median[IQR] | 29.783[28.511,30.833] | 29.589[27.681,30.990] | 29.810[28.628,30.814] | <0.001 |
| HGB/PLT ,median[IQR] | 0.545[0.450,0.653] | 0.490[0.386,0.597] | 0.556[0.464,0.662] | <0.001 |
| PLT/RBC ,median[IQR] | 53.378[44.850,64.303] | 59.059[48.565,73.012] | 52.596[44.211,62.712] | <0.001 |
| PLR ,median[IQR] | 121.667[97.122,158.503] | 142.697[108.532,193.478] | 118.878[95.588,152.273] | <0.001 |
| NLR ,median[IQR] | 1.979[1.485,2.826] | 2.357[1.686,3.518] | 1.922[1.462,2.694] | <0.001 |
| NLR/PLT ,median[IQR] | 500.824[352.364,763.200] | 606.797[385.359,967.026] | 485.116[347.898,728.406] | <0.001 |
| LYMPH/MONO ,median[IQR] | 4.944[3.556,6.320] | 4.257[3.046,5.816] | 5.065[3.688,6.400] | <0.001 |
| NEUT/MONO ,median[IQR] | 9.786[8.021,11.978] | 10.091[7.947,12.778] | 9.738[8.031,11.854] | <0.001 |
| LDL/HDL ,median[IQR] | 2.153[1.647,2.805] | 2.153[1.625,2.413] | 2.163[1.650,2.876] | <0.001 |
| MONO/HDL ,median[IQR] | 0.307[0.223,0.426] | 0.307[0.230,0.381] | 0.307[0.222,0.434] | 0.003 |
| FHDL ,median[IQR] | 3.810[3.130,4.550] | 3.810[3.140,4.100] | 3.820[3.130,4.620] | <0.001 |
| CHOL/HDL ,median[IQR] | 3.720[3.051,4.576] | 3.720[3.060,4.040] | 3.730[3.048,4.679] | <0.001 |
| TG/HDL ,median[IQR] | 1.398[0.938,2.124] | 1.328[0.798,1.409] | 1.441[0.968,2.238] | <0.001 |
| Urea/Crea ,median[IQR] | 0.068[0.057,0.081] | 0.077[0.068,0.097] | 0.068[0.056,0.077] | <0.001 |
| UA/Crea ,median[IQR] | 4.427[3.461,5.885] | 4.427[3.317,5.639] | 4.427[3.511,5.931] | <0.001 |
| UA/HDL ,median[IQR] | 251.956[145.976,367.123] | 237.533[142.935,251.956] | 260.736[146.927,388.095] | <0.001 |
| UA/LYMPH ,median[IQR] | 172.678[102.893,249.749] | 159.064[94.224,229.577] | 175.686[104.405,253.297] | <0.001 |
| NLR/PLR ,median[IQR] | 0.016[0.013,0.021] | 0.017[0.013,0.021] | 0.016[0.013,0.021] | 0.346 |
| CRP/CHOL ,median[IQR] | 0.591[0.377,1.890] | 0.591[0.482,2.478] | 0.571[0.370,1.816] | <0.001 |

**Supplementary Table S2. Univariate analysis of candidate variables.**

| **Variable** | **N** | **OR** | **95%CI** | ***P*-value** |
| --- | --- | --- | --- | --- |
| age | 9903.0 | 0.969 | [0.966,0.972] | 0.000 |
| WBC | 9903.0 | 1.078 | [1.052,1.105] | 0.000 |
| BASO | 9903.0 | 812736.318 | [51232.449,12893007.009] | 0.000 |
| NEUT | 9903.0 | 0.998 | [0.973,1.023] | 0.850 |
| MONO | 9903.0 | 1.69 | [1.253,2.279] | 0.001 |
| LYMPH | 9903.0 | 2.054 | [1.887,2.235] | 0.000 |
| EO | 9903.0 | 72976539641.766 | [548457799.518,9710091355743.887] | 0.000 |
| BASO rate | 9903.0 | 1.878 | [1.549,2.277] | 0.000 |
| NEUT rate | 9903.0 | 0.97 | [0.965,0.975] | 0.000 |
| MONO rate | 9903.0 | 0.947 | [0.919,0.977] | 0.000 |
| LYMPH rate | 9903.0 | 1.033 | [1.027,1.039] | 0.000 |
| EO rate | 9903.0 | 7.63 | [5.069,11.485] | 0.000 |
| RBC | 9903.0 | 3.016 | [2.772,3.281] | 0.000 |
| HGB | 9903.0 | 1.047 | [1.044,1.05] | 0.000 |
| HCT | 9903.0 | 12404154.306 | [4309418.265,35703901.224] | 0.000 |
| MCV | 9903.0 | 1.008 | [1.001,1.015] | 0.028 |
| MCH | 9903.0 | 1.056 | [1.038,1.073] | 0.000 |
| MCHC | 9903.0 | 1.029 | [1.025,1.034] | 0.000 |
| RDWCV | 9903.0 | 0.798 | [0.775,0.82] | 0.000 |
| RDWSD | 9903.0 | 0.905 | [0.895,0.916] | 0.000 |
| PLT | 9903.0 | 0.999 | [0.998,1.0] | 0.010 |
| LDL | 9903.0 | 1.218 | [1.152,1.288] | 0.000 |
| UA | 9903.0 | 1.003 | [1.003,1.003] | 0.000 |
| CHOL | 9903.0 | 1.126 | [1.081,1.173] | 0.000 |
| Urea | 9903.0 | 1.017 | [0.994,1.04] | 0.159 |
| Crea | 9903.0 | 1.032 | [1.029,1.036] | 0.000 |
| TG | 9903.0 | 1.494 | [1.409,1.584] | 0.000 |
| CO2 | 9903.0 | 1.008 | [0.999,1.018] | 0.083 |
| HDL | 9903.0 | 0.926 | [0.83,1.034] | 0.171 |
| RF | 9903.0 | 0.954 | [0.951,0.957] | 0.000 |
| ASO | 9903.0 | 1.002 | [1.001,1.003] | 0.000 |
| CRP | 9903.0 | 0.997 | [0.995,0.998] | 0.000 |
| ESR | 9903.0 | 0.987 | [0.985,0.989] | 0.000 |
| WBC/PLT | 9903.0 | 19510780.799 | [61089.461,6231362319.579] | 0.000 |
| WBC/RBC | 9903.0 | 0.777 | [0.717,0.841] | 0.000 |
| WBC/HGB | 9903.0 | 0.0 | [0.0,0.001] | 0.000 |
| HGB/RBC | 9903.0 | 1.056 | [1.038,1.073] | 0.000 |
| HGB/PLT | 9903.0 | 15.538 | [10.846,22.262] | 0.000 |
| PLT/RBC | 9903.0 | 0.981 | [0.978,0.983] | 0.000 |
| PLR | 9903.0 | 0.995 | [0.994,0.996] | 0.000 |
| NLR | 9903.0 | 0.934 | [0.917,0.952] | 0.000 |
| NLR/PLT | 9903.0 | 1.0 | [1.0,1.0] | 0.000 |
| LYMPH/MONO | 9903.0 | 1.125 | [1.096,1.155] | 0.000 |
| NEUT/MONO | 9903.0 | 0.99 | [0.984,0.997] | 0.004 |
| CHOL/TG | 9903.0 | 0.785 | [0.759,0.812] | 0.000 |
| LDL/HDL | 9903.0 | 1.278 | [1.203,1.358] | 0.000 |
| MONO/HDL | 9903.0 | 1.813 | [1.374,2.393] | 0.000 |
| FHDL | 9903.0 | 1.181 | [1.129,1.236] | 0.000 |
| CHOL/HDL | 9903.0 | 1.183 | [1.132,1.236] | 0.000 |
| TG/HDL | 9903.0 | 1.304 | [1.237,1.375] | 0.000 |
| Urea/Crea | 9903.0 | 0.0 | [0.0,0.0] | 0.000 |
| UA/Crea | 9903.0 | 1.047 | [1.025,1.07] | 0.000 |
| UA/HDL | 9903.0 | 1.002 | [1.002,1.002] | 0.000 |
| UA/LYMPH | 9903.0 | 1.001 | [1.001,1.001] | 0.000 |
| NLR/PLR | 9903.0 | 3.201 | [0.01,1052.157] | 0.694 |
| CRP/CHOL | 9903.0 | 0.996 | [0.99,1.001] | 0.115 |
| sex |  |  |  |  |
| Female | 3645.0 |  |  |  |
| Male | 6258.0 | 7.779 | [6.876,8.801] | 0.000 |

**Supplementary Table S3. Multicollinearity diagnosis of candidate features using Variance Inflation Factor (VIF) analysis.**

| **Variable** | **VIF** |
| --- | --- |
| MCH | inf |
| HGB/RBC | inf |
| WBC | 41526.267 |
| NEUT | 33574.315 |
| CHOL | 6152.666 |
| FHDL | 5242.844 |
| LYMPH | 3806.169 |
| NEUT rate | 1210.323 |
| LYMPH rate | 1181.503 |
| HCT | 1156.901 |
| HGB | 1048.934 |
| MCV | 960.843 |
| HDL | 631.903 |
| EO rate | 324.565 |
| MONO | 276.799 |
| NLR/PLR | 238.754 |
| WBC/PLT | 226.501 |
| MCHC | 182.196 |
| RBC | 100.587 |
| WBC/RBC | 98.833 |
| WBC/HGB | 96.307 |
| EO | 55.861 |
| MONO rate | 49.104 |
| PLT/RBC | 43.279 |
| PLT | 41.086 |
| NLR/PLT | 36.761 |
| CHOL/HDL | 33.048 |
| LDL/HDL | 24.761 |
| NLR | 24.382 |
| LDL | 21.622 |
| BASO | 21.422 |
| HGB/PLT | 18.756 |
| PLR | 18.615 |
| BASO rate | 16.106 |
| RDWCV | 13.624 |
| TGHDL | 12.991 |
| TG | 12.070 |
| LYMPH/MONO | 11.052 |
| RDWSD | 9.520 |
| MONO/HDL | 8.031 |
| CRP | 6.435 |
| CRP/CHOL | 6.372 |
| UA/HDL | 5.813 |
| UA/LYMPH | 5.757 |
| NEUT/MONO | 3.153 |
| Urea | 2.908 |
| UA/Crea | 2.691 |
| Urea/Crea | 2.639 |
| Crea | 2.572 |
| UA | 2.423 |
| CHOL/TG | 2.241 |
| sex | 1.955 |
| age | 1.789 |
| ESR | 1.647 |
| RF | 1.142 |
| ASO | 1.059 |
| CO2 | 1.039 |

**Supplementary Table S4. LASSO regression coefficients.**

| **Name** | **Minimum Mean Square Error Coefficient** | **Standard Error Coefficient of Minimum Distance** |
| --- | --- | --- |
| (Intercept) | -1.375 | -1.908 |
| sex | 1.482 | 1.459 |
| age | -0.001 | 0.0 |
| WBC | -0.12 | -0.041 |
| BASO | -2.491 | -1.676 |
| NEUT | 0.0 | 0.0 |
| MONO | 0.532 | 0.0 |
| LYMPH | -0.04 | -0.071 |
| EO | 0.0 | 0.0 |
| BASO rate | 0.019 | 0.0 |
| NEUT rate | 0.0 | 0.0 |
| MONO rate | -0.084 | -0.047 |
| LYMPH rate | 0.022 | 0.018 |
| EO rate | 0.372 | 0.252 |
| RBC | -0.836 | -0.218 |
| HGB | -0.033 | 0.0 |
| HCT | 31.891 | 13.433 |
| MCV | -0.037 | 0.0 |
| MCHC | 0.005 | -0.005 |
| RDWCV | -0.017 | -0.034 |
| MCH | 0.0 | -0.008 |
| RDWSD | -0.059 | -0.049 |
| PLT | 0.001 | 0.001 |
| LDL | -0.06 | -0.001 |
| UA | 0.001 | 0.001 |
| CHOL | -29.571 | -18.754 |
| Urea | 0.087 | 0.09 |
| Crea | 0.008 | 0.007 |
| TG | 0.113 | 0.168 |
| CO2 | 0.007 | 0.006 |
| HDL | 30.079 | 19.384 |
| RF | -0.04 | -0.039 |
| ASO | 0.0 | 0.0 |
| CRP | -0.002 | -0.006 |
| ESR | 0.003 | 0.003 |
| WBC/PLT | -18.074 | 0.0 |
| WBC/RBC | 0.606 | 0.27 |
| WBC/HGB | -3.31 | 0.0 |
| HGB/RBC | 0.0 | 0.0 |
| HGB/PLT | -0.551 | -0.762 |
| PLT/RBC | -0.001 | 0.0 |
| PLR | -0.002 | -0.002 |
| NLR | 0.009 | 0.0 |
| NLR/PLT | 0.0 | 0.0 |
| LYMPH/MONO | 0.0 | 0.006 |
| NEUT/MONO | 0.005 | 0.003 |
| CHOL/TG | -0.15 | -0.116 |
| LDL/HDL | 0.0 | 0.0 |
| MONO/HDL | 0.246 | 0.276 |
| FHDL | 29.544 | 18.634 |
| CHOL/HDL | 0.052 | 0.118 |
| TG/HDL | -0.118 | -0.139 |
| Urea/Crea | -4.829 | -5.191 |
| UA/Crea | -0.001 | 0.0 |
| UA/HDL | 0.0 | 0.0 |
| UA/LYMPH | 0.001 | 0.001 |
| NLR/PLR | 18.599 | 0.0 |
| CRP/CHOL | 0.029 | 0.05 |

**Supplementary Table S5. Performance comparison of different machine learning models in the training set.**

| Models | AUC(95%CI) | cutoff(95%CI) | Accuracy(95%CI) | Sensitivity(95%CI) | Specificity(95%CI) | PPV(95%CI) | NPV(95%CI) | F1-score(95%CI) | Kappa(95%CI) |
| --- | --- | --- | --- | --- | --- | --- | --- | --- | --- |
| XGBoost | 0.937(0.930-0.945) | 0.764(0.764-0.764) | 0.892(0.892-0.892) | 0.905(0.905-0.905) | 0.829(0.829-0.829) | 0.964(0.964-0.964) | 0.635(0.635-0.635) | 0.933(0.933-0.933) | 0.654(0.654-0.654) |
| logistic | 0.872 (0.861-0.883) | 0.825(0.825-0.825) | 0.769(0.769-0.769) | 0.76(0.760-0.760) | 0.815(0.815-0.815) | 0.954(0.954-0.954) | 0.404(0.404-0.404) | 0.846(0.846-0.846) | 0.409(0.409-0.409) |
| LightGBM | 0.717 (0.703-0.731) | 0.925(0.925-0.925) | 0.881(0.881-0.881) | 0.962(0.962-0.962) | 0.472(0.472-0.472) | 0.901(0.901-0.901) | 0.715(0.715-0.715) | 0.931(0.931-0.931) | 0.503(0.503-0.503) |
| RandomForest | 0.998 (0.998-0.999) | 0.64(0.640-0.640) | 0.978(0.978-0.978) | 0.974(0.974-0.974) | 0.999(0.999-0.999) | 1.0(1.000-1.000) | 0.883(0.883-0.883) | 0.987(0.987-0.987) | 0.924(0.924-0.924) |
| AdaBoost | 0.913 (0.905-0.922) | 0.507(0.507-0.507) | 0.866(0.866-0.866) | 0.886(0.886-0.886) | 0.767(0.767-0.767) | 0.95(0.950-0.950) | 0.573(0.573-0.573) | 0.917(0.917-0.917) | 0.575(0.575-0.575) |
| DecisionTree | 0.922 (0.915-0.929) | 0.837(0.837-0.837) | 0.85(0.850-0.850) | 0.859(0.859-0.859) | 0.805(0.805-0.805) | 0.957(0.957-0.957) | 0.532(0.532-0.532) | 0.905(0.905-0.905) | 0.551(0.551-0.551) |
| GBDT | 0.905 (0.896-0.914) | 0.872(0.872-0.872) | 0.807(0.807-0.807) | 0.801(0.801-0.801) | 0.838(0.838-0.838) | 0.961(0.961-0.961) | 0.456(0.456-0.456) | 0.874(0.874-0.874) | 0.479(0.479-0.479) |
| GNB | 0.860 (0.848-0.872) | 0.883(0.883-0.883) | 0.79(0.790-0.790) | 0.791(0.791-0.791) | 0.785(0.785-0.785) | 0.948(0.948-0.948) | 0.428(0.428-0.428) | 0.863(0.863-0.863) | 0.432(0.432-0.432) |
| SVM | 0.481 (0.460-0.502) | 0.814(0.800-0.828) | 0.723(0.702-0.743) | 0.791(0.764-0.818) | 0.378(0.366-0.391) | 0.864(0.863-0.866) | 0.268(0.246-0.291) | 0.826(0.811-0.841) | 0.146(0.125-0.168) |

**Note:** **Abbreviations:** PPV, positive predictive value; NPV, negative predictive value; AUC, area under the curve.

**Supplementary Table S6. Construction and evaluation of the optimal model test set**

| AUC(95%CI) | cutoff | Accuracy | Sensitivity | Specificity | Positive Predictive Value | Negative Predictive Value | F1-score | Kappa |
| --- | --- | --- | --- | --- | --- | --- | --- | --- |
| 0.898 (0.879-0.917) | 0.509 | 0.827 | 0.834 | 0.794 | 0.954 | 0.485 | 0.89 | 0.5 |

**Supplementary Table S7. Baseline demographic and clinical characteristics of patients in the independent historical validation cohort.**

| Variable | Rheumatoid Arthritis (n=171) | Gouty Arthritis (n=1812) | P |
| --- | --- | --- | --- |
| Sex ,n(%) |  |  | <0.001 |
| Female | 127(74.269) | 673(37.141) |  |
| Male | 44(25.731) | 1139(62.859) |  |
| UA ,median[IQR] | 325.000[248.000,382.000] | 438.100[371.200,533.000] | <0.001 |
| Crea ,median[IQR] | 64.840[53.780,79.810] | 78.000[66.000,91.260] | <0.001 |
| RF ,median[IQR] | 45.400[16.600,133.400] | 6.100[2.900,12.200] | <0.001 |
| ESR ,median[IQR] | 47.000[18.000,79.000] | 23.000[11.000,42.000] | <0.001 |
| CHOL/TG ,median[IQR] | 3.646[2.743,5.387] | 2.902[2.003,4.219] | 0.014 |

**Supplementary Table S8.** **Internal time verification test set**

| AUC | cutoff | Accuracy | Sensitivity | Specificity | Positive Predictive Value | Negative Predictive Value | F1-score | Kappa | AUC_L | AUC_U |
| --- | --- | --- | --- | --- | --- | --- | --- | --- | --- | --- |
| 0.897 | 0.509 | 0.767 | 0.759 | 0.848 | 0.981 | 0.249 | 0.856 | 0.291 | 0.871 | 0.924 |

**Supplementary Figure1:**

**Supplementary Figure S1. Performance evaluation and learning diagnostics of the AdaBoost model.** (A) ROC curve for the training set, showing a discriminative AUC of 0.926. (B) ROC curve for the validation set, demonstrating robust generalization with an AUC of 0.897. (C) Learning curve analysis. The convergence of training (red dashed line) and validation (blue dashed line) scores indicates minimal overfitting and adequate sample size for model stability. (D) Confusion matrix heatmap (Training set). Rows and columns represent true and predicted classes (0/1), respectively. Color intensity reflects sample counts per category.
